# Crystalline Peroxosolvates: Nature of the Coformer, Hydrogen-Bonded Networks and Clusters, Intermolecular Interactions

**DOI:** 10.3390/molecules26010026

**Published:** 2020-12-23

**Authors:** Alexander G. Medvedev, Andrei V. Churakov, Petr V. Prikhodchenko, Ovadia Lev, Mikhail V. Vener

**Affiliations:** 1 Kurnakov Institute of General and Inorganic Chemistry, Russian Academy of Sciences, Leninskii Prosp. 31, 119991 Moscow, Russia; medvedev.chem@gmail.com (A.G.M.); churakov@igic.ras.ru (A.V.C.); prikhman@gmail.com (P.V.P.); 2 The Casali Center of Applied Chemistry, The Institute of Chemistry, The Hebrew University of Jerusalem, Jerusalem 91904, Israel; 3 Department of Quantum Chemistry, Mendeleev University of Chemical Technology, Miusskaya Square 9, 125047 Moscow, Russia

**Keywords:** two-component crystals, isomorphous H_2_O_2_/H_2_O substitution, periodic DFT computations, hydrogen bond enthalpy and energy, peroxosolvates of high-energy compounds, mixed pharmaceutical forms, hydrogen peroxide

## Abstract

Despite the technological importance of urea perhydrate (percarbamide) and sodium percarbonate, and the growing technological attention to solid forms of peroxide, fewer than 45 peroxosolvates were known by 2000. However, recent advances in X-ray diffractometers more than tripled the number of structurally characterized peroxosolvates over the last 20 years, and even more so, allowed energetic interpretation and gleaning deeper insight into peroxosolvate stability. To date, 134 crystalline peroxosolvates have been structurally resolved providing sufficient insight to justify a first review article on the subject. In the first chapter of the review, a comprehensive analysis of the structural databases is carried out revealing the nature of the co-former in crystalline peroxosolvates. In the majority of cases, the coformers can be classified into three groups: (1) salts of inorganic and carboxylic acids; (2) amino acids, peptides, and related zwitterions; and (3) molecular compounds with a lone electron pair on nitrogen and/or oxygen atoms. The second chapter of the review is devoted to H-bonding in peroxosolvates. The database search and energy statistics revealed the importance of intermolecular hydrogen bonds (H-bonds) which play a structure-directing role in the considered crystals. H_2_O_2_ always forms two H-bonds as a proton donor, the energy of which is higher than the energy of analogous H-bonds existing in isostructural crystalline hydrates. This phenomenon is due to the higher acidity of H_2_O_2_ compared to water and the conformational mobility of H_2_O_2_. The dihedral angle H-O-O-H varies from 20 to 180° in crystalline peroxosolvates. As a result, infinite H-bonded 1D chain clusters are formed, consisting of H_2_O_2_ molecules, H_2_O_2_ and water molecules, and H_2_O_2_ and halogen anions. H_2_O_2_ can form up to four H-bonds as a proton acceptor. The third chapter of the review is devoted to energetic computations and in particular density functional theory with periodic boundary conditions. The approaches are considered in detail, allowing one to obtain the H-bond energies in crystals. DFT computations provide deeper insight into the stability of peroxosolvates and explain why percarbamide and sodium percarbonate are stable to H_2_O_2_/H_2_O isomorphic transformations. The review ends with a description of the main modern trends in the synthesis of crystalline peroxosolvates, in particular, the production of peroxosolvates of high-energy compounds and mixed pharmaceutical forms with antiseptic and analgesic effects.

## 1. Introduction

Crystalline peroxosolvates, adducts of hydrogen peroxide, were first introduced by Tanatar who synthesized sodium percarbonate Na_2_CO_3_·1.5H_2_O_2_ [1] and urea perhydrate (percarbamide) CH_4_N_2_O·H_2_O_2_ [2]. These compounds are the two most widely used solid peroxocompounds with annual production in the millions of tons [3]. Sustainable, nontoxic, and minimal hazard processing trends combine to intensify the use of hydrogen peroxide in diverse fields and the same trends are responsible for the perpetually growing use of peroxosolvates [3,4,5]. Peroxosolvates are now used for bleaching, disinfection, and oxidation; as chemical reagents in household commodities, cosmetics, pharmaceuticals, and washing powders; in industrial environmental processes such as remediation, bioremediation, and oxygen production; and as explosive ingredients and reagents for chemical synthesis [3,4,5,6]. In general, hydrogen peroxide release from peroxosolvates tends to lower the pH, whereas hydroperoxo- and peroxo-complexes tend to increase the pH [6,7,8,9,10]. Thus, peroxosolvates are considered safer and more economic as aqueous hydrogen peroxide decomposes at high pH.

From an academic point of view, the insight gained from the energetics of peroxosolvates as reflected by single crystal x-ray studies and DFT computations sheds light on the non-redox behavior of hydrogen peroxide in aqueous and biological systems. Selective transmembrane uptake and transport, bioactivation, and detoxification of hydrogen peroxide are all likely to involve non-redox bonding mechanisms, which also determine crystalline peroxosolvate formation and stability in aqueous media [11,12,13]. It is not a mere coincidence that one of the largest classes of peroxosolvates involves amino acids and dipeptides. In fact, the number of structurally resolved amino acid peroxosolvates outweighs the number of the corresponding hydrates. Peroxosolvates of 14 amino acids are structurally characterized, while only six of these amino acids form hydrates according to the Cambridge Structural Database (CSD) [14,15]. The ability to form networks of strong intermolecular hydrogen bonds (H-bonds) both in solution and in the crystalline phase determines the key role that H_2_O_2_ plays in ecologically significant and biological processes [16,17,18]. Hydrogen peroxide is formed in living cells in mitochondria [11,19]. H_2_O_2_ plays an important role in oxidative stress processes [18,20]. Several integral membrane proteins act as transmembrane channels promoting the hydrogen peroxide transport across cell membranes [21,22,23,24].

The H-bond is the main type of intermolecular interaction in crystalline peroxosolvates. The hydrogen peroxide molecule is capable of forming up to six such bonds: two as a proton donor and four as an acceptor [25]. However, at the moment, only a few crystalline peroxosolvates are known, in which the hydrogen peroxide molecule forms six H-bonds [25,26,27]. Hydrogen peroxide has pronounced acidic properties, which, for example, are manifested in the ability to deprotonate under mild conditions and form ammonium hydroperoxide [28] and metal peroxides [29]. We have previously shown that, due to its pronounced acidic properties, the hydrogen peroxide molecule in peroxosolvates always forms two H-bonds as a proton donor, which are structure-directing [25]. This suggests that the compounds forming stable peroxosolvates should contain proton-acceptor groups, that is, they are Brønsted bases or have amphoteric properties.

CSD [14,15] and the Inorganic Crystal Structure Database (ICSD) [30,31] contain information on 134 peroxosolvates. This is several orders of magnitude less than the number of crystalline hydrates that existed in these databases about twenty years ago [32]. As a result of the analysis of structural databases, the chemical composition and networks of H-bonds in peroxosolvates, in which H_2_O_2_ molecules do not directly interact with metal atoms, have been characterized [25]. The study of more than 260 H-bonds in 65 crystal structures showed that hydrogen peroxide always participates as proton donor in two H-bonds and forms from zero to four hydrogen bonds as a proton acceptor.

**Figure 1:**
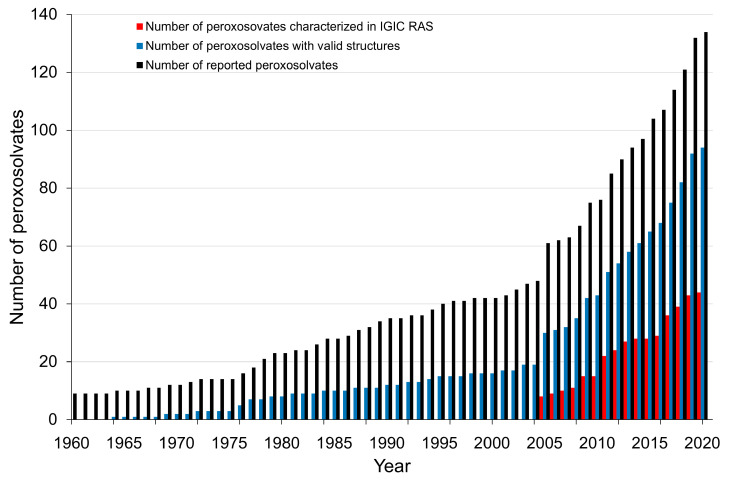
Cumulative chart of the total number of peroxosolvates in the period 1960–2020.

Taking into account the peroxosolvates synthesized over the past four years, as well as crystals with an H_2_O_2_–metal atom contact [33,34,35], the total number of crystalline peroxosolvates is 134. This is two times more than the number of crystal structures analyzed in [25]. A significant number of peroxosolvates (44 adducts) were synthesized and structurally characterized at the Kurnakov Institute of General and Inorganic Chemistry of Russian Academy of Sciences (IGIS RAS) (Figure 1).

The search for new peroxosolvates is an active task. Analysis of the composition and structure of known crystalline peroxosolvates made it possible to formulate the main directions of such a search, which are outlined at the end of Section 2.

The presented material is arranged as follows. First, the chemical composition of crystalline peroxosolvates is considered. This made it possible to formulate criteria for the directed synthesis of new stable crystalline H_2_O_2_ adducts with certain properties: mixed pharmaceutical forms, high-energy substances, etc. Section 3 is devoted to the dimension and topology of peroxide clusters existing in crystalline peroxosolvates. The main focus is on infinite one-dimensional (1D) chains of H_2_O_2_ molecules. Then, approaches based on calculations using density functional theory methods with periodic boundary conditions are considered, which make it possible to obtain the energies of intermolecular H_2_O_2_ interactions in organic crystals. The fifth section describes the specific features of the H-bond networks in crystalline peroxosolvates. Particular attention is paid to the analysis of the lengths of H-bonds formed by H_2_O_2_ as a proton acceptor, and the types of H_2_O_2_ coordination. The review ends with a description of the main modern trends in the synthesis of crystalline peroxosolvates.

## 2. Chemical Composition of Crystalline Peroxosolvates

**Figure 2:**
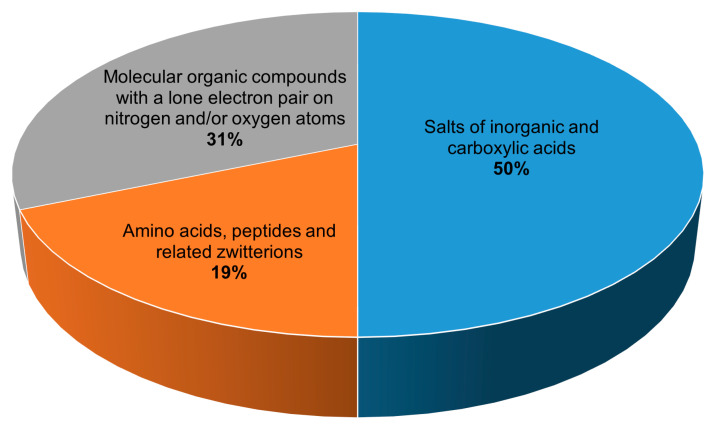
Distribution of peroxosolvates by the chemical nature of the coformer.

All 134 crystalline peroxosolvates known to date can be divided into three main groups depending on the chemical nature of the coformer (Figure 2):

(1) salts of inorganic and carboxylic acids,

(2) amino acids, peptides and related zwitterions, and 

(3) molecular compounds with a lone electron pair on nitrogen and/or oxygen atoms.

Intermolecular H-bonds play a structure-directing role in the considered crystals.

The largest number of structurally characterized peroxosolvates, 67 compounds, are adducts of hydrogen peroxide and salts of inorganic and carboxylic acids with various cations. Among the salts of carboxylic acids that form stable peroxosolvates [36,37,38,39,40], oxalates can be distinguished in the composition of six compounds. The salts of inorganic acids that form adducts with hydrogen peroxide are very diverse. In addition to fluorides [41,42,43], chlorides, and bromides [44,45], a number of peroxosolvates of alkali metal and ammonium carbonates are known [46,47,48]. The latter include the commercially demanded sodium peroxocarbonate synthesized by Tanatar [1]. This class of compounds should include peroxosolvates of complex anions, which can formally be attributed to the salts of the corresponding complex acids, for example, peroxovanadates [49,50,51,52,53,54,55,56], peroxoniobates [57,58,59], peroxotantalates [60], uranyl peroxo complexes [61], peroxotellurates [62], and platinum complexes [63,64,65]. Peroxosolvates of metal peroxides [66,67,68,69] can formally belong to the specified class of peroxosolvates of salts of inorganic acids if hydrogen peroxide is considered as a diacid.

The next group in terms of the number of compounds (42 compounds) are peroxosolvates formed by molecular organic compounds with a lone electron pair(s) on the nitrogen and/or oxygen atom(s). The main representatives of this group of crystalline hydrogen peroxide adducts are organophosphorus compounds containing the P=O functional group [49,70,71,72,73,74,75,76] and nitrogen-containing heterocyclic compounds [25,26,77,78,79,80,81,82,83,84], in particular N-oxides [85,86,87,88,89,90,91,92], obtained as a result of the oxidation reaction of the corresponding compounds with hydrogen peroxide. Urea peroxosolvate [2,27] is used as a solid source of hydrogen peroxide, and, along with 1,4-diazabicyclo[2.2.2]octane (DABCO) peroxosolvate [93], is used in organic syntheses to obtain anhydrous hydrogen peroxide solutions.

Separately, it is worth highlighting the third group, which includes amino acids, peptides, and related zwitterions (25 peroxosolvates). Peroxosolvates of a number of proteionogenic l-amino acids (serine, threonine, leucine, isoleucine, tyrosine, glycine, and phenylalanine) [94,95] and non-proteinogenic amino acids (gamma-aminobutyric acid, beta-alanine, and sarcosine) were obtained and structurally characterized at the Kurnakov Institute of General and Inorganic Chemistry RAS [95,96]. The class of zwitterions related to amino acids that form peroxosolvates includes pyridine carboxylic acids: nicotinic, isonicotinic, and picolinic acids [97], as well as 2-aminonicotinic acid [85]. Peroxosolvates of cyclic dipeptides—diglycine, disarcosine, and dialanine—are an example of the nonoxidative interaction of concentrated hydrogen peroxide and a peptide fragment [13].

The CSD analysis revealed the necessary properties of co-former peroxosolvates, which are promising compounds for the synthesis of new crystalline peroxosolvates [25]:

(1) Hydrogen peroxide should not participate in redox reactions with coformers.

(2) They must be sufficiently soluble in protic solvents to carry out the crystallization process.

(3) Coformers should have the ability to form H-bonds, primarily as proton acceptors. 

(4) Compounds with pronounced acidic properties do not form peroxosolvates [14], since in such compounds the proton-acceptor groups are protonated.

(5) Coformers should exhibit amphoteric or basic properties. Strong bases deprotonate hydrogen peroxide and form peroxide or hydroperoxide as ionic or complex moieties (ZnO_2_ [29], NH_4_^+^OOH^−^ [28,98], or [Sn(OOH)_6_]^2−^ [7]).

A significant part of the 134 peroxosolvates available in the structural databases, namely 40 structures, contain incomplete or erroneous data. Some crystal structures contain unlocalized hydrogen atoms [39,51,55,57,99,100,101,102,103] and errors in the O-O bond lengths [56,104] and H-O-O-H and O-O-H angles [105,106] in the hydrogen peroxide molecule. This is due to the fact that during the preparation of these compounds, H_2_O_2_ was used as an oxidizing agent or ligand to obtain the corresponding peroxo complexes; therefore, the mass content of hydrogen peroxide in the obtained crystals is low. The chemical composition of 94 crystal structures of peroxosolvates with objectively localized protons and free of structural errors is presented in Supplementary Materials Table S1. These 94 crystal structures are the subject of this review, as they allow one to analyze the topology of H_2_O_2_ hydrogen-bonded networks.

## 3. Dimensions and Topology of Peroxide Clusters in the Crystalline Phase

It was already mentioned above that peroxosolvates exist due to a system of various H-bonds formed by hydrogen peroxide with coformers in crystals. The question arises, can H-bonds between hydrogen peroxide molecules, in addition to H-bonds with organic molecules, be observed in the structures of crystalline peroxosolvates? Furthermore, if so, what are the dimensions and topology of the formed peroxide clusters? 

Over the past 10 years, a number of structures have been described containing insular (finite) clusters of two H_2_O_2_ molecules in peroxosolvates of peroxovanadates [56,107] and in potassium alumoxalate peroxalate [108]. A linear centrosymmetric cluster of three hydrogen peroxide molecules [72] with the D3 topology in the Infantes–Motherwell notation [109] was obtained. In the potassium peroxocarbonate peroxosolvate, a cyclic cluster consisting of four hydrogen peroxide molecules was found [110]. Somewhat later, another example of a dimeric cluster was found in the structure of tyrosine peroxosolvate [95]. More recently, data on the structures of lidocaine N-oxide and 2-aminonicotinic acid peroxosolvates were published, including stellar pentameric and giant dodecameric clusters of peroxide molecules [85]. The last of these clusters cannot be accurately classified within the Infantes–Motherwell notation and its topology can be seen as a combination of R4 and D5 motifs.

**Figure 3:**
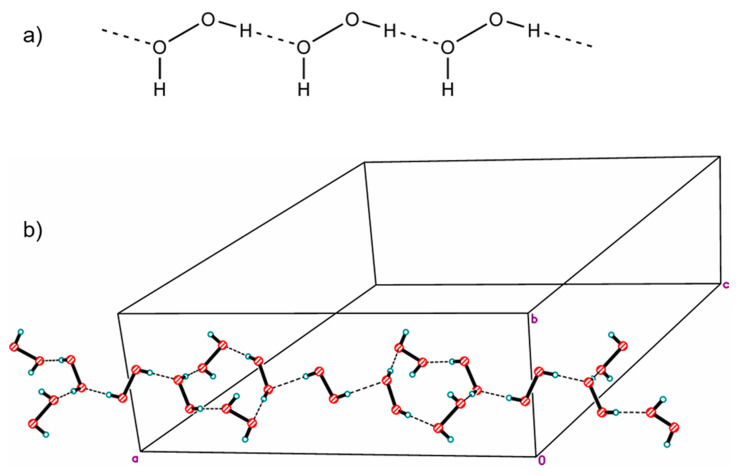
Infinite one-dimensional chains of hydrogen peroxide molecules: (**a**) the simplest topology C1 (YAFGEU) and (**b**) a nontrivial topology, which is a combination of alternating linear and cyclic fragments (SEMXIU). H-bonds are denoted by dotted lines.

In 1984, the structure of a mixed hydrate of a peroxosolvate with infinite one-dimensional chains of H_2_O_2_ molecules with the simplest topology C1 was published. In the chain, some H_2_O_2_ molecules were statistically replaced by water molecules due to the phenomenon of mutual isomorphic substitution of peroxide and water molecules, recently studied in the work [25]. Subsequently, the same peroxide–water chains were found in the structures of thymine peroxosolvate hydrates [111] and DABCO [93]. In these three cases, the presence of an impurity of water is explained by the fact that the authors carried out crystallization from dilute solutions of hydrogen peroxide in water. Only in 2017 was anhydrous thymine peroxosolvate (from 98% peroxide) containing “pure peroxide” C1 chains obtained (Figure 3a) [25].

Two more examples of “purely peroxide” chains of trivial C1 topology were recently discovered in the structures of phenylserine and pipicolinic acid peroxosolvates [96,112]. An example of the structure of an organic peroxosolvate with infinite peroxide chains of a nontrivial topology was presented in [80], which is a combination of alternating linear and cyclic fragments consisting only of H_2_O_2_ molecules (Figure 3b).

**Figure 4:**
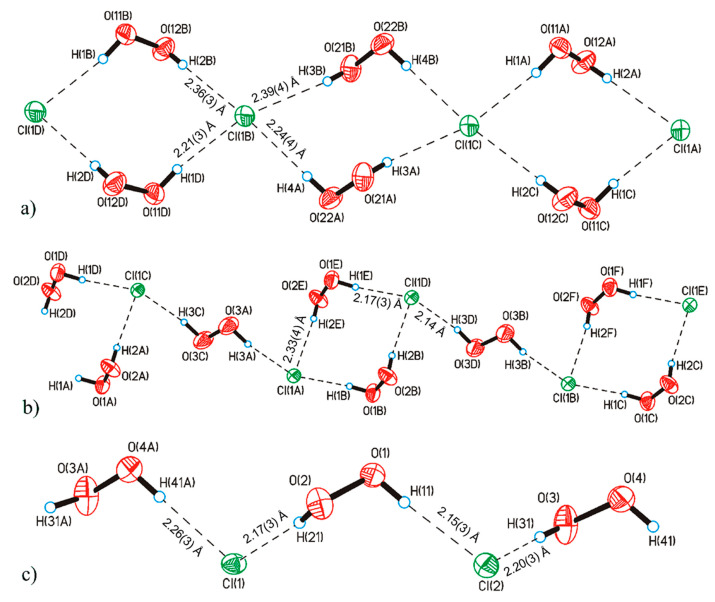
Structure of hydrogen peroxide clusters formed in crystalline Ph_4_AsCl·*n*H_2_O_2_: (**a**) [(H_2_O_2_)_2_(Cl^−^)_1_]_inf_ chains at *n* = 2; (**b**) [(H_2_O_2_)_3_(Cl^−^)_2_]_inf_ chains at *n* = 1.5; (**c**) [(H_2_O_2_)_1_(Cl^−^)_1_]_inf_ chains at *n* = 1. O-H···Cl^−^ bonds are denoted by dotted lines. Distances H···Cl^−^ are presented. Reproduced from the work in [45] with permission from The Royal Society of Chemistry.

It should be noted that hydrogen peroxide tends to form infinite one-dimensional H-bonded chain clusters with halogen anions (Figure 4). This phenomenon was studied in detail in [45], which led to the discovery of the phenomenon of peroxomorphism by the example of crystallization of solvatomorphs with the composition (Ph_4_As)^+^Cl^−·^*n*H_2_O_2_ (*n* = 1, 1.5, 2) from peroxide solutions of different concentrations (30, 50, and 96%).

## 4. The Energies of Intermolecular Interactions of H_2_O_2_ in Organic Crystals: Calculations by the Kohn–Sham Methods with Periodic Boundary Conditions

Various theoretical approaches/methods are used to describe the structure and properties of organic crystals: calculations in the cluster approximation [113], methods using empirical force fields (molecular dynamics) [114,115], and calculations by the Kohn–Sham methods with periodic boundary conditions (periodic DFT) [116,117]. Calculations in the cluster approximation can deduce certain properties of an organic crystal: the energy of the crystal lattice [118], the mobility of charges in organic semiconductors [119,120], the chemical shift of the nucleus [121]. This requires knowledge of the structure of the crystal under study, that is, the cif file. Empirical force field parameters have limited transferability [122]. Simultaneous calculation of various properties of an organic crystal (energy of intermolecular interactions, enthalpy of sublimation, low-frequency IR and Raman spectra, etc.) requires the use of periodic DFT methods [123,124]. There are a number of methods and programs for performing this type of calculation [125,126]. In computational methods of solid-state physics, approaches that utilize basic sets of plane waves are usually used [117], and in solid state chemistry, basic sets of the Gaussian type are used [116]. The advantages and disadvantages of these approaches in calculating organic crystals are discussed in [124,125,127].

In the theoretical study of crystals, much attention is paid to assessing the energy of intermolecular interactions. In contrast to the gas phase, such a calculation in a solid is a non-trivial problem, which involves the “isolation” of the energy (enthalpy) of a specific intermolecular interaction from the energy of the crystal lattice (enthalpy of sublimation) [128]. In most cases, empirical approaches are used that relate the energy of intermolecular interaction with one or another parameter of the electron density at the bond critical point [129,130,131]. In this case, the calculated values of the electron density, the values of the parameters retrieved from the precise X-ray diffraction data, and hybrid approaches are used [132]. (Calculation of the electron density characteristics in plane wave basis is not trivial due to the use of pseudopotentials [133].) The approaches indicated above are often used to assess intermolecular interactions of various natures in various crystals [134,135,136], which gives rise to well-founded criticism [137,138].

Intermolecular interactions of H_2_O_2_ in crystalline peroxosolvates are mainly due to conventional H-bonds. The energy or enthalpy of these bonds can be estimated from the spectroscopic [139] and metric [140] characteristics of H-bonds in crystals, that is, without invoking the electron density parameters. 

The H-bond enthalpy (−Δ*H_HB_*) can be estimated as previously shown [140]:

−Δ*H_HB_* [kJ/mol] = 0.134·*R*(H···B)^−3.05^. (1)

Here, the *R*(H···B) is the H···B distance (nm), and B = O, N. The main limitation of this approach is the problem of accurate experimental determination of the position of hydrogen atoms. (As noted above, many papers have been published in which the position of hydrogen atoms in crystalline peroxosolvates has not been determined.) For this it is necessary to use the neutron diffraction method. We note that the number of crystals with H-bonds studied by this method is very limited [141]; in particular, this method was used to study the H_2_O_2_ crystal [142]. The exact values of the H···B distances can be computed using the periodic DFT methods [117,125].

The −Δ*H_HB_* value can be evaluated using Equation (2) [139]:

−Δ*H_HB_* [kJ/mol] = 1.386·(Δν [cm^−1^] − 40)^0.5^, (2)

here Δν = ν(OH_free_) − ν(OH); ν(OH_free_) and ν(OH) are the frequencies of free and H-bonded OH group stretching vibrations, respectively [133]. They can be determined both experimentally and by periodic DFT calculations.

The H-bond energy, *E_HB_*, is calculated as [129]

*E_HB_* [kJ/mol] = 1124·*G_b_* [a.e.]. (3)

Here, *G_b_* is local electronic kinetic energy density at the bond critical point. It can be calculated by periodic DFT methods or obtained using Kirzhnitz’s approximation from experimental data [143].

Comparison of enthalpies/energies of intermolecular H-bonds in crystals of organic molecules, in particular, crystalline peroxosolvates, obtained using approximations (1), (2), and (3) was carried out in a number of papers [13,144,145,146]. Significant differences in the calculated values are observed only for short (strong) H-bonds [147], which are caused by the contribution of the covalent component to the energy of these bonds [148,149]. These approaches yield energies/enthalpies of weak and moderate hydrogen bonds that are in good agreement with each other. Thus, Equation (3) gives reasonable values of the energies of intermolecular interactions driven by the electrostatic factor, that is, for weak and moderate H-bonds and nonconventional H-bonds. It can be recommended for evaluating the energy of intermolecular H-bonds in crystals.

There are also other schemes that make it possible to accurately calculate the enthalpy [139] and energy [143] of intermolecular H-bonds in crystals. However, the first approach uses the integral intensity of the stretching vibration of the O-H group, which is extremely difficult to measure experimentally for an arbitrary organic crystal, and the second approach is limited to the O-H···O fragment.

## 5. Examples of H-bond Networks: Average Distances, Types of Coordination

**Figure 5:**
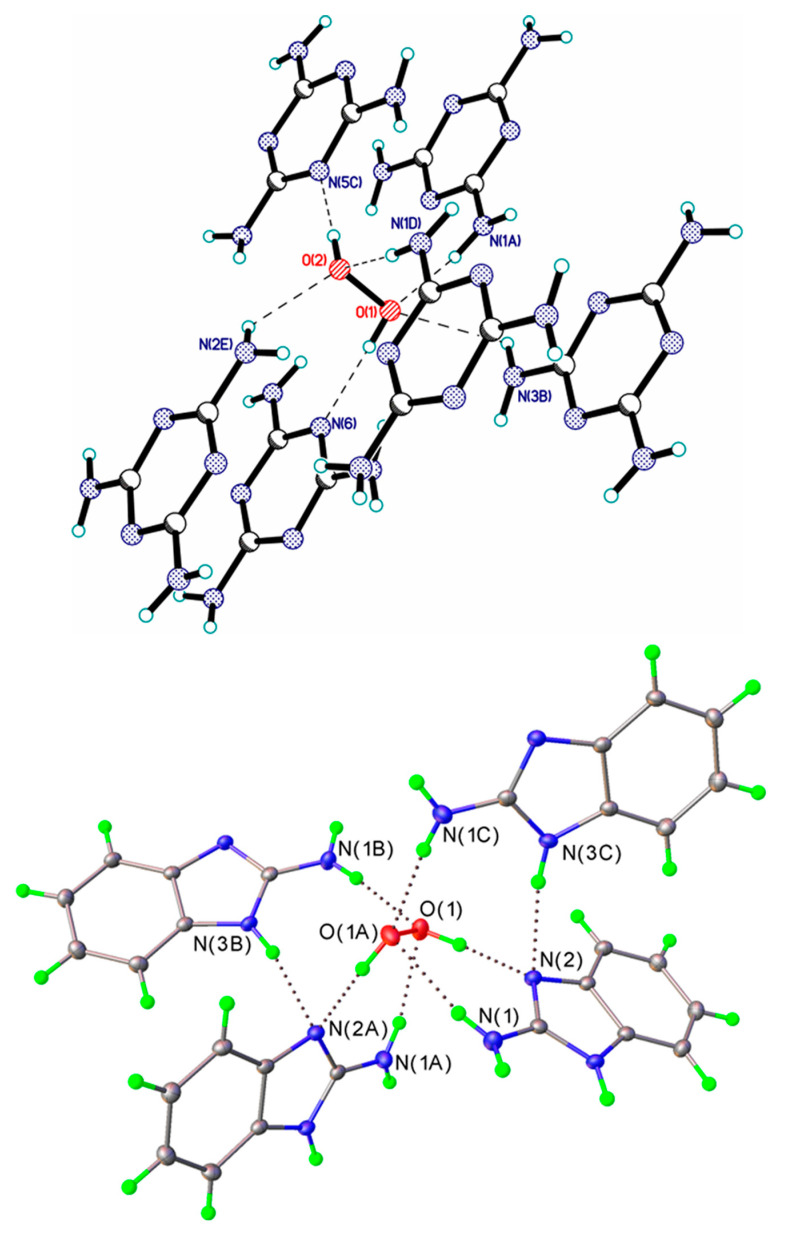
Crystals with the maximum number of H-bonds. H-bonds are denoted by dotted lines. Top panel: melamine peroxosolvate. Adapted with permission from the authors of [25]. Copyright 2017 American Chemical Society. Bottom panel—2-aminobenzimidazole peroxosolvate. Reproduced from the work in [26] with permission from The Royal Society of Chemistry.

**Figure 6:**
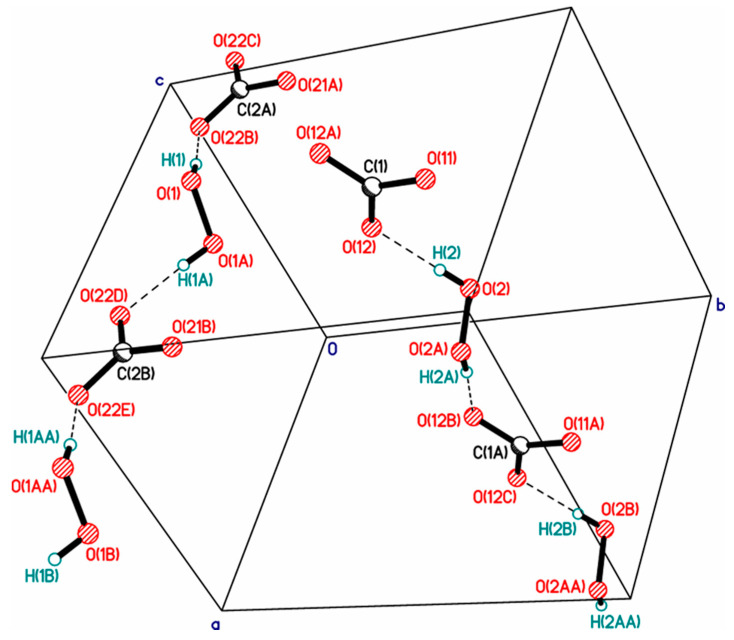
Fragment of the crystal structure of ammonium carbonate peroxosolvate, in which the H_2_O_2_ molecule forms only two H-bonds, as a proton donor; they are given by dotted lines. Reproduced with permission from the work in [48]. Copyright 2012 International Union of Crystallography.

In currently known crystalline peroxosolvates, the number of hydrogen peroxide molecules in the asymmetric unit of the crystal structures varies within wide range: ¼ [65], ½ [92], 1 [144], 1½ [150], 2 [45], 3 [96], and 6 [85]. The following general rules were formulated [25]. (1) Hydrogen peroxide acts as a proton donor in two H-bonds. (2) H_2_O_2_ forms from 0 to 4 hydrogen bonds as a proton acceptor (Figure 5); however, there are crystals in which these bonds are absent (Figure 6). (3) The total number of H-bonds varies from two to six. In most crystals, H_2_O_2_ forms one or two H-bonds as a proton acceptor, while three or four such H-bonds are realized very rarely (Figure 5). According to work [151], this is explained by the insufficient amount of acidic protons in most organic coformers. Urea perhydrate [27] has long been known as the only crystal in which the H_2_O_2_ molecule interacts through six H-bonds with the surrounding organic coformers. In recent years, two additional crystals have been structurally characterized at the Kurnakov Institute of General and Inorganic Chemistry RAS in which H_2_O_2_ also forms the maximum number of H-bonds: melamine peroxosolvate C_3_H_6_N_6_ H_2_O_2_ [25] and 2-aminobenzimidazole peroxosolvate 2(C_7_H_7_N_3_) H_2_O_2_ [26] (Figure 5). To search for new stable and inexpensive peroxosolvates with a high active oxygen content (Section 6), the organic coformers of these crystals must have a ratio of proton-donor groups to groups with lone electron pairs equal to 2 [26]. Note that the coformers of melamine and 2-aminobenzimidazole peroxosolvates, as well as urea, have an NH_2_ group, that is, this rule is fulfilled.

**Figure 7:**
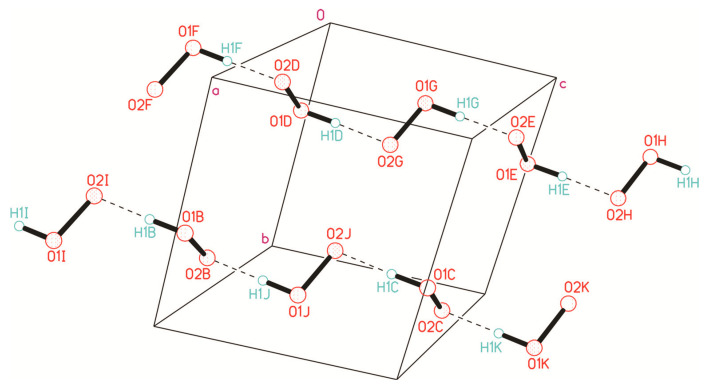
Infinite ionic chains in the NH_4_^+^OOH^−^ crystal, consisting of HOO^−^ fragments interacting through short (strong) H-bonds, which are given by dashed lines. Reproduced with permission from J. Chem. Phys. 133, 16 (2010). Copyright 2004 AIP Publishing [28].

Let us begin to consider the networks of H-bonds formed by H_2_O_2_ in crystalline peroxosolvates with H-bonds as a proton donor, as they are realized in all crystals. [25,26]. Moderate O-H···O^−^ bonds are usually formed in amino acid peroxosolvates [85,94,95,96,152]; here, O^−^ denotes the oxygen of the CO_2_**^−^** group of the amino acid zwitterion. These are the so-called charge-assisted H-bonds [153,154]. Distances *d*(O···O^−^) range from 2.604 to 2.776 Å [95], and the angle O-H···O^−^ is usually more than 160°. The indicated values of the O···O^−^ distances practically do not differ from the distances in the neutral O-H···O bonds in hydrogen peroxide dihydrate [155] and phenylserine peroxosolvate [96]. A different situation is realized in the case of anionic chains of hydrogen peroxide [25] (Figure 7). These chains are stabilized by short (strong) H-bonds [156]. The *d*(O···O^−^) distance is ~2.53 Å [98], and the O-H···O^−^ angle is 178° [28].

**Figure 8:**
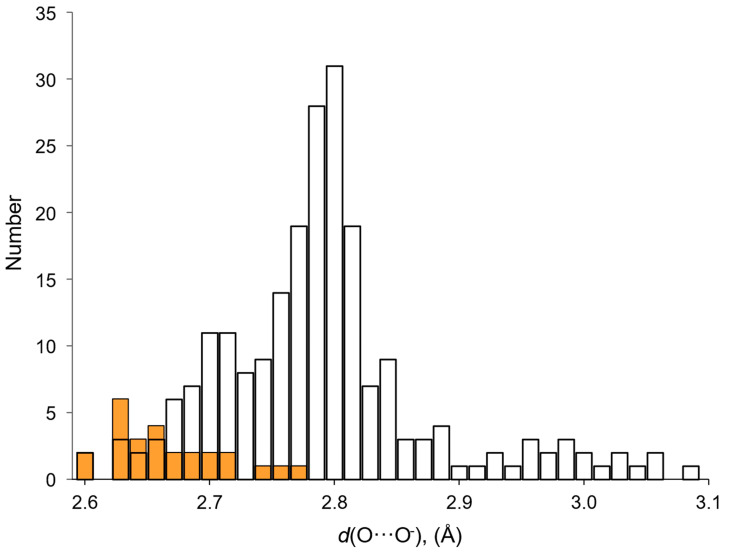
Distribution of O···O^−^ distances *d*(O···O^−^) in H-bonds formed by hydrogen peroxide and water molecules as proton donors in amino acid crystals. White bars—crystalline hydrates; orange bars—peroxosolvates.

The features of the considered H-bonds in perhydrates are clearly manifested when comparing the networks of H-bonds in crystalline peroxosolvates and amino acid hydrates. Currently, nine crystals of natural amino acid peroxosolvates containing 26 H-bonds as a proton donor have been characterized (Table 1) and a large number of α-amino acid hydrates (not necessarily natural) containing 221 such H-bonds. The average value of the distance *d*(O···O^−^) in peroxosolvates of amino acids is 2.67 Å (Table 1), which is much less than the analogous value in hydrates—2.80 Å [14,15]. This is due to the presence in hydrates of a large number of H-bonds with distances *d*(O···O^−^) > 2.8 Å (Figure 8), while in peroxosolvates the maximum value of this distance is 2.776 Å (Table 1). The *d*(O···O) distances in “neutral” H-bonds in amino acid peroxosolvates practically do not differ from the corresponding *d*(O···O^−^) values in “charge-assisted” H-bonds [94,95].

In mixed halogen-peroxide chains having O–H···Cl^−^ and O–H···Br^−^ bonds (Figure 4), the shortest O-H···Cl^−^/O-H···Br^−^ distances are 3.02/3.19 Å [45]. According to the data in Table 4 of the review [157], such a value of *d*(O···Cl^−^) corresponds to the shortest O-H···Cl^−^ bonds in crystal hydrates.

Thus, the distances *d*(O···X), where X = O, O^−^, Cl^−^, etc., in the intermolecular H-bonds formed by H_2_O_2_ molecules as proton donors are systematically shorter than similar bonds formed by water molecules. According to Equation (1), this means that the enthalpy of H-bonds formed by H_2_O_2_ is greater than the values of −Δ*H_HB_* for bonds formed by water molecules. Calculations of the enthalpies/energies of H-bonds by Equations (2) and (3) confirm this conclusion [13,146,158]. This phenomenon can be explained by the higher acidity of H_2_O_2_ in comparison with water, see introduction, and the conformational mobility of the H_2_O_2_ molecule. In contrast to water in crystalline peroxosolvates, the dihedral angle between OH groups in H_2_O_2_ is usually ~90° [142]. 

**Figure 9:**
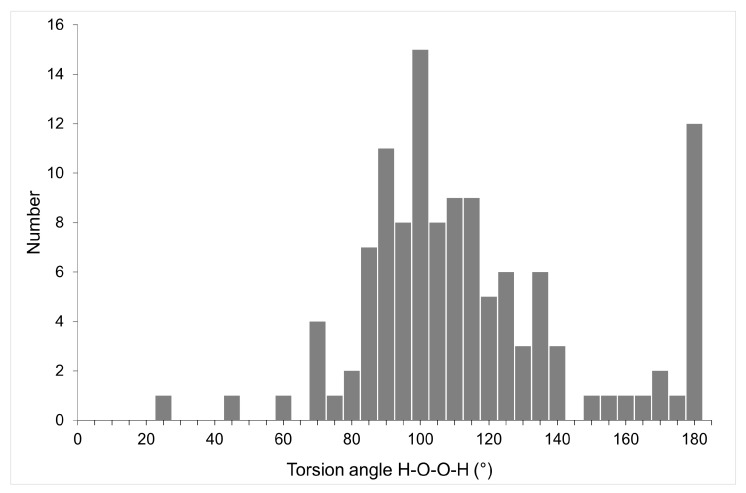
Distribution of H-O-O-H torsion angles in crystalline peroxosolvates.

Hydrogen peroxide is the simplest nonplanar molecule, and its geometry is determined by the H-O-O-H torsion angle. In the gas phase, according to IR and microwave spectroscopy data, the most stable conformation is characterized by a torsion angle of 119.8° [159]. The H_2_O_2_ molecule easily rotates around the central O–O bond with *cis* and *trans* barriers 7.0 and 1.1 kcal/mol [160]. In crystalline hydrogen peroxide, the torsion angle is, according to X-ray diffraction and neutron diffraction data, 90.2(6) and 94(2)°, respectively [142,161]. The experimentally found values of torsion angles in crystalline peroxosolvates, according to the latest version of CSD, occupy the entire range of values from 0 to 180° with maxima of about 95 and 180° (Figure 9). According to this figure, some peroxosolvates have a HOOH torsion of about 180°. This phenomenon can be explained by the effects of crystal packing, see below.

It should be noted that the state of these torsion angles in the crystalline phases is somewhat different from the gaseous and liquid phases, as hydrogen peroxide in crystals can be located both in general positions (without symmetry elements) and in special positions (at the centers of inversion i, 2-fold rotation axis and mirror planes m). Axes 2 do not impose restrictions on the values of the H-O-O-H angles; planes m are not realized in the structures of organic peroxosolvates. In nine structures (CAZHUH, GADOXP10, KUMRER, VAYGUY01, BAFJUQ, VAYMAJ, YUHTAW, ZUWCIG, and KELXEH) peroxide molecules lie on crystallographic centers i with rigidly fixed torsion angles of 180°.

**Figure 10:**
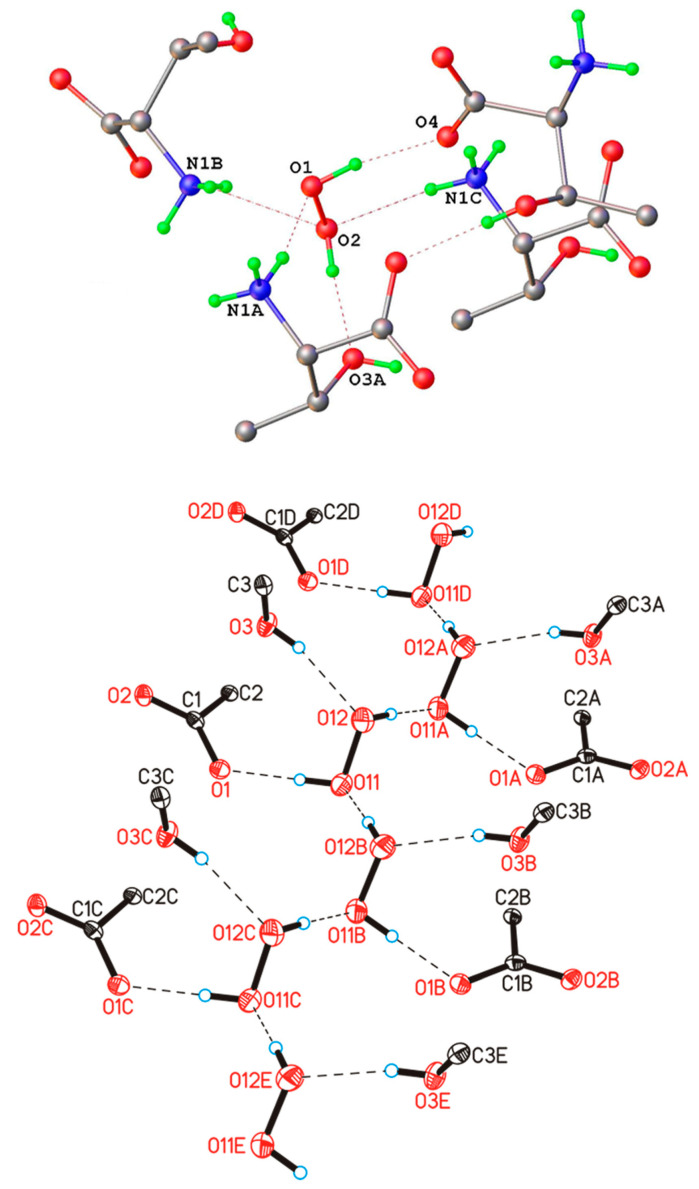
H-bond networks in amino acid peroxosolvates. Upper panel: l-threonine peroxosolvate: three H-bonds between the H_2_O_2_ molecule and the NH_3_^+^ groups. (Reproduced from the work in [95] with permission from The Royal Society of Chemistry) Lower panel: phenylserine peroxosolvate: no H-bonds between the H_2_O_2_ molecule and the NH_3_^+^ groups. The phenyl and amino groups of phenylserine are omitted. (Reproduced from the work in [96] with permission from The Royal Society of Chemistry). H-bonds are shown with dashed lines.

The number, type, and strength of H-bonds formed by the H_2_O_2_ molecule in peroxosolvates as a proton acceptor are larger and more diverse than the analogous characteristics/properties of H-bonds, in which H_2_O_2_ acts as a proton donor. This is due to two reasons: First, the number of “acceptor” H-bonds can vary from 0 to 4 (Figure 5 and Figure 6). Second, the type and strength of such H-bonds are determined by the nature and charge of the proton-donor group of the coformer. In the case of natural amino acids, the considered H-bonds are mainly due to the interaction of the lone electron pair H_2_O_2_ with the NH_3_^+^ group of the amino acid (Table 2). Distances d(O···N^+^) vary from ~2.64 [162] to ~2.83 Å [25], however, both “short” and “long” O···HN^+^ bonds can be highly nonlinear, with the O···H-N^+^ angle less than 140°. Usually, one or two H-bonds are formed, while peroxosolvates of natural amino acids with three H-bonds or without H-bonds between the H_2_O_2_ and NH_3_^+^ group of the amino acid (Figure 10) are much less common (Table 2). The latter case is realized when H_2_O_2_ molecules form endless chains of H-bonds (Figure 10). As a result, the crystal may lack H-bonds between the H_2_O_2_ and NH_3_^+^ group of the amino acid [96].

**Figure 11:**
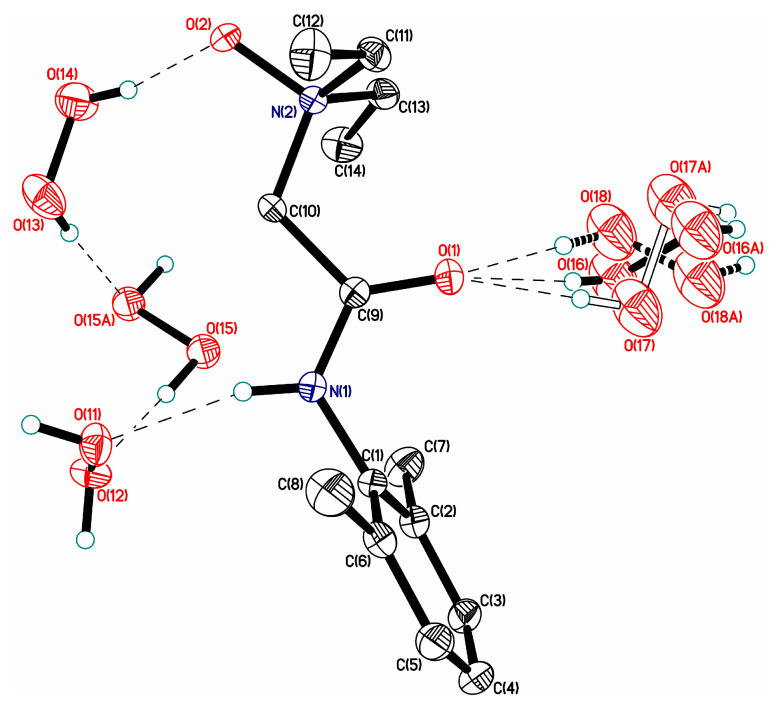
Neutral H-bonds between the NH group and hydrogen peroxide in the crystal of the peroxosolvate lidocaine N-oxide C_14_H_22_N_2_O_2_·3H_2_O_2_. H-bonds are given by dotted lines. Reprinted with permission from the authors of [85]. Copyright 2017 WileyVCH Verlag GmbH & Co. KGaA.

The NH_2_ groups of the co-former usually form “neutral” H-bonds with hydrogen peroxide. These bonds are relatively weak [157] and are characterized by distances *d*(N···O) > 3.0 Å [25,27]. In the presence of “peptide” groups, such as –CONH [13] or the NH-group of picolinic acid [97], the formation of “neutral” H-bonds between these groups and hydrogen peroxide is possible (Figure 11). In a number of peroxosolvates of natural amino acids, neutral O···H-O bonds are realized, where, for example, O-H is the hydroxyl group of l-threonine (Figure 10).

**Figure 12:**
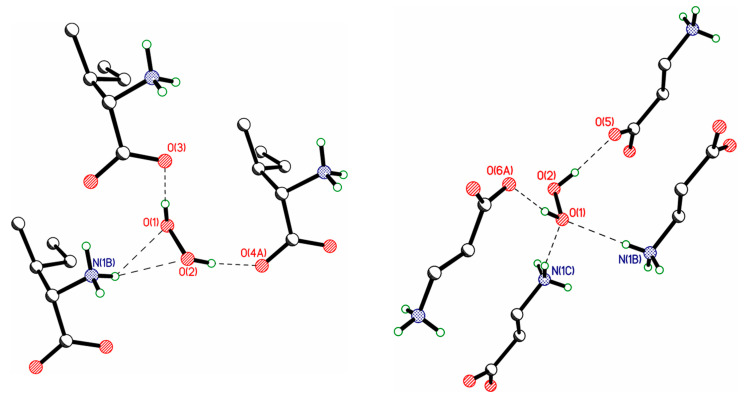
Bifurcate H-bonds, which are realized in isoleucine (TANDET, left panel) and β-alanine (TANDAP, right panel) peroxosolvates.

**Figure 13:**
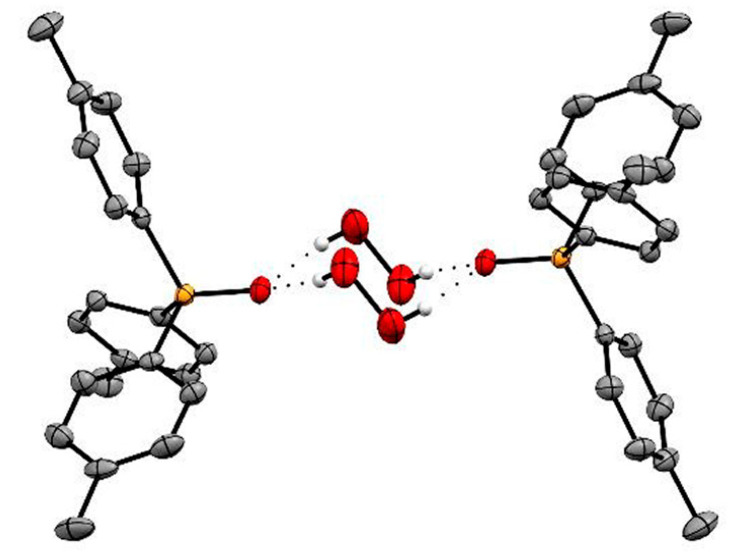
Single crystal structure of (p-Tol_3_PO·H_2_O_2_)_2_. Reproduced from the work in [71] with permission from The Royal Society of Chemistry.

**Figure 14:**
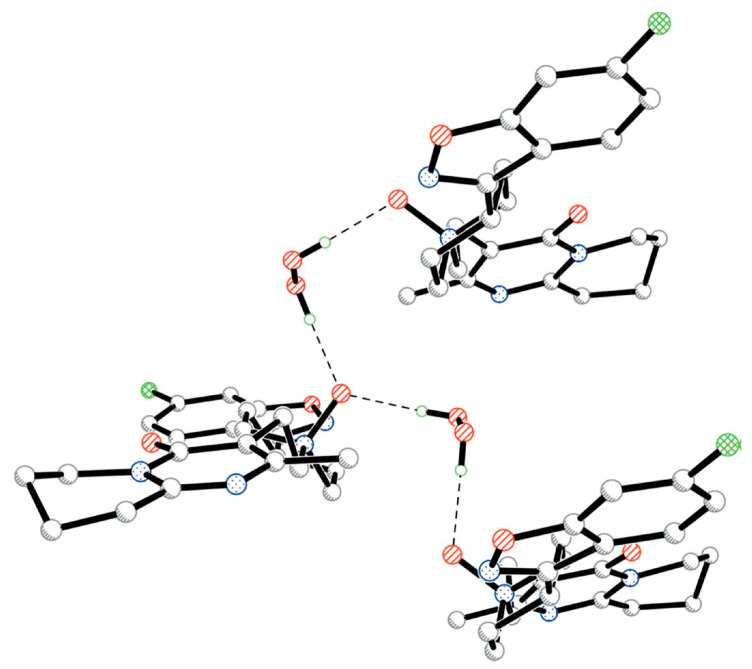
Fragment of the crystal structure of risperidone N-oxide, illustrating the interaction of hydrogen peroxide with coformer molecules through O-H···O bonds (dashed lines). H atoms attached to C atoms have been omitted for clarity. Reproduced with permission from the authors of [86]. Copyright 2005 International Union of Crystallography.

**Figure 15:**
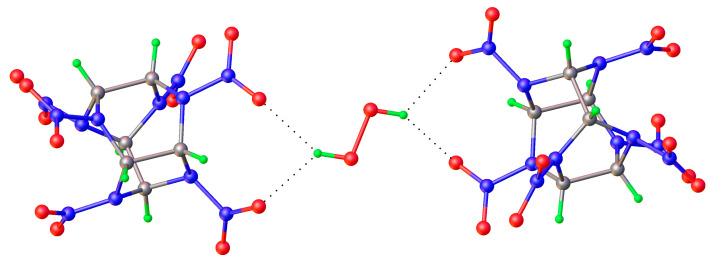
Bifurcate H-bond in 2,4,6,8,10,12-hexanitro-2,4,6,8,10,12-hexaazoisowurtzitane peroxosolvate [84].

In the literature, much attention is paid to bifurcated H-bonds, see Section 3.4 in [157]. In contrast to the C=O and P=O groups, which quite often form such bonds [163,164], the oxygen atoms of hydrogen peroxide rarely participate in bifurcate H-bonds. Two examples of such H-bonds are shown in Figure 12. On the other hand, coformers with P=O and N=O groups and not containing active (acidic) hydrogen atoms form bifurcated H-bonds with H_2_O_2_ molecules [71,86] (Figure 13 and Figure 14). In these structures, H_2_O_2_ molecules usually form only two H-bonds, as proton donors. However, H_2_O_2_ clusters are also realized, in which some H_2_O_2_ molecules interact with each other, with one of the hydrogen peroxide molecules acting as a proton acceptor [72,85]. At the moment, there is one example of a bifurcate H-bond formed by a hydrogen peroxide molecule and a nitro group 2,4,6,8,10,12-hexanitro-2,4,6,8,10,12-hexaazoisowurtzitane [84] in one of the polymorphs of the crystalline peroxosolvate of this molecule (Figure 15).

In organic crystals, nonconventional O···H-C [128] bonds often arise. In crystalline peroxosolvates, such bonds are rarely formed [165].

Note that in the case of coformers, the size of which significantly exceeds the size of a hydrogen peroxide molecule, H-bonds can arise between the coformers (Figure 10, upper panel). This increases the already wide variety of H-bond networks found in crystalline peroxosolvates. 

In addition to the formation of H-bonds with anions of inorganic and organic acids, hydrogen peroxide molecules form contacts with alkali metals, enabling additional stabilization of the peroxosolvate. The contact distances between the alkali metal (Li, Na, K, Rb, and Cs) and the oxygen atom of the H_2_O_2_ molecule are shown in Table S1. When searching for distances, the following distance constraints were used: Li-O (2.5 Å), Na-O (3.0 Å), K/Rb/Cs-O (3.5 Å). Analysis of similar contacts between an alkali metal and an oxygen atom of a water molecule in hydrates according to CSD data did not reveal significant differences between the values of the analyzed distances in peroxosolvates and hydrates. The average contact distances in hydrates and peroxolvates were 1.97, 2.42, 2.88, 3.06, and 3.22 Å for Li, Na, K, Rb, and Cs, respectively. The Zn-O distance in the only structurally characterized molecular zinc complex Zn^II^(H_2_O_2_) is 2.172 Å, while the hydrogen peroxide molecule also participates in the formation of two H-bonds as a proton donor [35]. Thus, hydrogen peroxide can be considered as a weak ligand, which manifests its coordination properties in a number of crystal structures of peroxosolvates due to relatively short contacts between oxygen atoms and the above alkali metal cations.

## 6. Trends and Prospects

### 6.1. High-Energy Substances

High-energy substances are a class of compounds that can generate large amounts of heat due to the extremely rapid exothermic decomposition reaction caused by external influences. Typical representatives of these compounds are nitroamines, for example, cyclotrimethylenetrinitramine [92] or nitro derivatives of triazole, for example, 1-methyl-3,5-dinitro-1,2,4-triazole [77], azasydnones [166], etc. High-energy density (i.e., high self-healing potential) in most high-energy substances usually leads to a decrease in stability and increased sensitivity to external influences. The main challenge in the production of new high-energy substances is to simultaneously achieve high energy density and stability to ensure safe production, storage and transportation. A promising method for solving this problem is the creation of two-component energy crystals, each component of which is a high-energy compound. The production of such crystals is difficult due to the poor “controllability” of the self-assembly process of the two components due to various intermolecular interactions (H-bonds, π-stacking, etc.). The synthesis of new two-component high-energy compounds requires the development of approaches and rules that make it possible to predict the self-assembly of molecules by isolating certain intermolecular interactions that form a structural motif in a crystal.

The concepts of “host” and “guest” (receptor and substrate), or the “key-and-lock” principle, underlie molecular recognition in supramolecular chemistry [167]. The effect of limited cavities in macrocyclic compounds—hosts (cyclodextrin, calixarene, cucurbituril, etc.)—allows them to recognize well small guest molecules with high binding energies [168]. The key-lock principle allows “controlling” the intermolecular interaction of various components and, thus, creating new high-energy substances.

**Figure 16:**
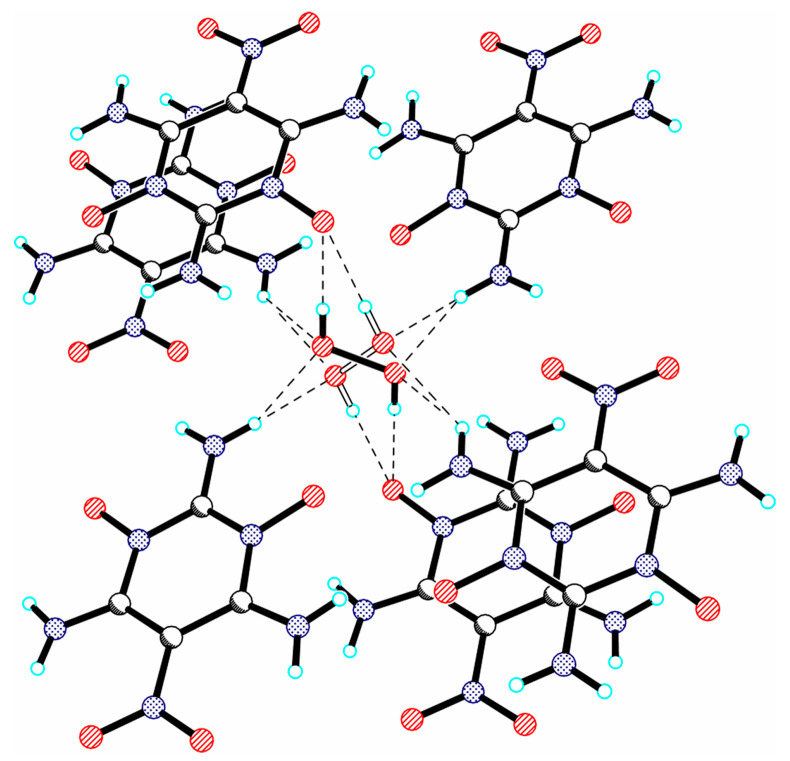
Fragment of the crystal structure of 2,4,6-triamino-5-nitropyrimidine-1,3-dioxide peroxosolvate, illustrating the interaction of H_2_O_2_ with surrounding host molecules through O-H···O and N-H···O bonds (dashed lines).

In [92], two-dimensional (2D) porous host materials were used to create energetic materials by trapping H_2_O_2_ (guest). Obviously, the sizes of cavities in porous 2D materials should correspond to the sizes of molecules with oxidizing properties. 2,4,6-Triamino-5-nitropyrimidine-1,3-dioxide (CM-102) has the ability to form H-bonds, primarily as proton acceptors. It was used as an energetic host, which has a 2D layered structure with cavities (Figure S1A in [92]). The H_2_O molecules are located between the ICM-102 layers, forming a sandwich structure (Figure S1A in [92]). It was found that H_2_O_2_ molecules replace water molecules in crystal hydrate CM-102 and are located in cavities, forming a graphite-like layered structure (Figure 2C in [92]). The peroxide molecule is crosswise disordered, and both components lie at the center of the inversion (Figure 16). H_2_O_2_ interacts with surrounding host molecules through O-H···O bonds with distances ~2.66 Å and N-H···O bonds with distances from 1.922 to 2.299 Å. The density of the obtained crystal was 1.915 g/cm^3^, that is, significantly higher than the density of crystalline hydrate CM-102, 1.845 g/cm^3^.

**Figure 17:**
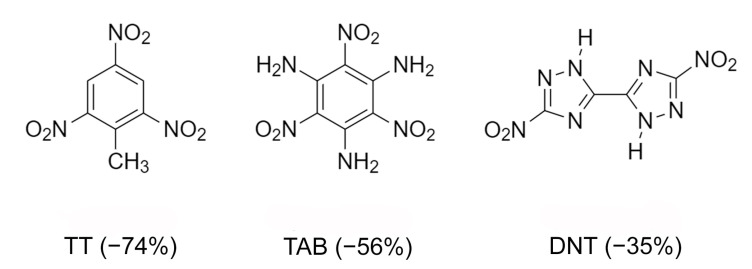
Chemical structure of 2,4,6-trinitrotoluene (TT), 2,4,6-triamino-1,3,5-trinitrobenzene (TAB) and DNT. Oxygen index of these compounds is given in parenthesis.

**Figure 18:**
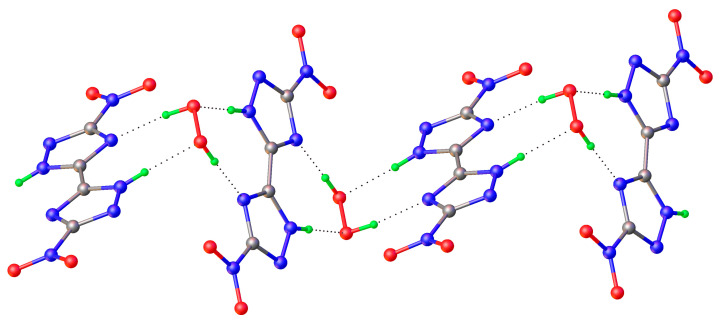
H-bond network in DNT crystal solvates [77].

A very important characteristic of high-energy substances is the so-called “oxygen index” [77]. It characterizes the mass percentage of oxygen available to oxidize conformer to neutral molecules. Usually energy materials are characterized by negative oxygen index values, that is, in most fuel cells there is an oxidant deficiency compared to the content of high-energy substances. Multicomponent crystallization can increase the oxygen index. Therefore, the preparation of crystalline peroxosolvates of high-energy substances is one of the ways to improve the value of the oxygen index [84]. The effect of polymorphism of peroxosolvates on the properties of high-energy compounds—azoles—was studied in [77]. 5,5’-Dinitro-2H,2H’-3,3’-bi-1,2,4-triazole (DNT) has been investigated because of its high detonation velocity and low impact sensitivity. DNT is characterized by an improved oxygen index (Figure 17). This compound has not found widespread use, in part because it does not form solvates under traditional crystallization conditions [169]. In [77], crystalline DNT hydrates were obtained; then, two crystalline peroxosolvates of this compound were synthesized. In both peroxosolvates, the H_2_O_2_ molecule forms four H-bonds with neighboring DNT molecules (Figure 18). The oxygen index in these peroxosolvates is −30%, which is significantly higher than the corresponding value for anhydrous DNT (−35%). Crystallization of peroxosolvates is an effective strategy for improving the performance of high-energy hydrate-prone materials.

The above approach was successfully used in [170] for the synthesis of a new H_2_O_2_-adduct of ammonium cyclopentazolate NH_4_N_5_∙½H_2_O_2_. This crystalline peroxosolvate is characterized by a high oxygen index (−22.86%) and a high calculated velocity and detonation pressure (8938 m/s and 26.37 GPa). H_2_O_2_ forms four H-bonds with the surrounding ions. The O-H···N bonds are almost linear (163°) and very short (2.811 Å) [170].

### 6.2. Mixed Pharmaceutical Forms. Antiseptic and Analgesic Effect

Pharmaceutical co-crystals and solvates are promising systems to improve the solubility of active pharmaceutical ingredients (API) [165,171]. H_2_O_2_ is green reagent, which is safe for human consumption. Therefore, hydrogen peroxide can be used as effective coformer.

**Figure 19:**
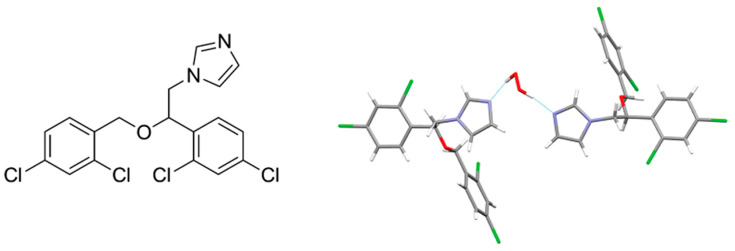
Chemical structure of miconazole (left). Asymmetric cell of the crystal structure of miconazole peroxosolvate [165].

Miconazole was chosen as the topical API (Figure 19). This compound is commonly used as a nitrate salt in ointments and powders, or in anhydrous form in gels and tablets. It also exists in the hydrated form from which the crystalline miconazole peroxosolvate was derived. There are two API molecules per H_2_O_2_ molecule in this crystal (Figure 19). H_2_O_2_ forms two N···H-O bonds with imidazole nitrogen atoms of two neighboring miconazole molecules with N...H distances equal to 1.75 and 1.81 Å. The corresponding values in the crystal hydrate of miconazole are slightly higher, 1.87 and 1.83 Å (see Section 5). Oxygen atoms of H_2_O_2_ and water molecules in both crystals do not form classic H-bonds, however, they interact with CH-groups of miconazole molecules through O···H-C bonds. The O···H distances are 2.23–2.50 Å for the peroxosolvate and 2.26–2.47 Å for the crystalline hydrate. The solubility of miconazole peroxosolvate in phosphate–citrate buffer at pH 4 was ~230 μg/mL, that is, significantly higher than the solubility of miconazole and its crystal hydrate, ~200 and ~160 μg/mL, respectively [165].

## 7. Conclusions

Water forms stable crystalline hydrates with almost all known classes of compounds. Hydrogen peroxide has significantly more pronounced acidic properties compared to water. Therefore, compounds with pronounced acidic properties do not form peroxosolvates. Compounds that can enter into redox reactions with hydrogen peroxide should also be excluded from the list of potential peroxosolvate coformers. These considerations may explain why the number of perhydrates is smaller than the number of known hydrates, but they cannot explain why the number of known crystalline peroxosolvates is several orders of magnitude smaller than that of known crystalline hydrates, which suggests that the chemistry of this class of compounds is seriously understudied.

A review of all currently known peroxosolvates allows us to unambiguously confirm the following general conclusions: (1) Every hydrogen peroxide molecule always participates as proton donor in two H-bonds; (2) Coformers should exhibit amphoteric or basic properties.

Analysis of structural databases revealed the nature of the molecules (coformers) that are prone to forming peroxosolvates. These are (a) salts of inorganic and carboxylic acids; (b) amino acids, peptides, and related zwitterions; and (c) molecular compounds with a lone electron pair on nitrogen and/or oxygen atoms. A promising method for the preparation of crystalline peroxosolvates is the replacement of a water molecule with H_2_O_2_ from structurally related solvates using a high concentration (>80%) of H_2_O_2_. Peroxosolvates can be obtained from aqueous hydrogen peroxide of low concentration (<30%) if their crystal structure meets one of the following requirements:

(1) H_2_O_2_ molecule forms at least five H-bonds with the surrounding molecules; (2) torsion angle of hydrogen peroxide molecule is close to 180°. Urea perhydrate and percabonate are fine demonstrations of these criteria.

## Figures and Tables

**Table 1:** H-bonds of H_2_O_2_ molecules as proton donors in crystalline peroxosolvates of amino acids and non-proteinogenic amino acids [95]: the number of H-bonds formed by one H_2_O_2_ molecule and O···O^−^ distances *d*(O···O^−^).

Crystal (Coformer)	The Number of H-Bonds	*d*(O⋯O^−^)/Å	
		1	2
l-Phenylalanine	2	2.634(2)	2.631(2)
l-Isoleucine	2	2.652(2)	2.634(2)
l-Isoleucine	2	2.707(2)	2.678(2)
2-Aminobutiric acid	2	2.697(1)	2.776(1)
	2	2.607(1)	2.664(1)
	2	2.682(1)	2.717(1)
l-Serine	1	2.706(2)	-
Glycine	2	2.648(1)	2.671(1)
	2	2.671(1)	2.636(1)
	2	2.645(1)	2.635(1)
l-Tyrosine	1	2.604(4)	2.760(3)
l-Threonine	1	2.637(2)	-
β-Alanine	2	2.666(1)	2.686(1)
	2	2.725(1)	2.753(1)
26 H-bonds		2.604–2.776	
Mean value		2.67	

**Table 2:** H-bonds of H_2_O_2_ molecules as proton acceptors in crystalline peroxosolvates of amino acids and non-proteinogenic amino acids [95]: the number of H-bonds formed by one H_2_O_2_ molecule and nature of the proton donor group.

Crystal (Coformer)	The Number of H-Bonds	The Proton Donor Group
l-Phenylalanine	2	–NH_3_^+^, –NH_3_^+^
l-Isoleucine	2	–NH_3_^+^, –NH_3_^+^
l-Isoleucine	1	–NH_3_^+^
2-Aminobutiric acid	2	–NH_3_^+^, –NH_3_^+^
	1	–NH_3_^+^
	1	–NH_3_^+^
l-Serine	2	–NH_3_^+^, –NH_3_^+^
Glycine	2	–NH_3_^+^, –NH_3_^+^
	2	–NH_3_^+^, –NH_3_^+^
	1	–NH_3_^+^
l-Tyrosine	2	–NH_3_^+^, H_2_O_2_
	1	–NH_3_^+^
l-Threonine	3	–NH_3_^+^, –NH_3_^+^, –NH_3_^+^
β-Alanine	2	–NH_3_^+^, –NH_3_^+^
	0	—

## Data Availability

The data presented in this study are available in Supplementary Materials.

## Supplementary Materials

The following are available online. Table S1: Structure features of peroxosolvates with localized protons, Table S2: Refcodes of proton disordered peroxosolvates.
