# Supplementary material for: Crystalline Peroxosolvates: Nature of the Coformer, Hydrogen-Bonded Networks and Clusters, Intermolecular Interactions
Source: Molecules. 2020 Dec 23;26(1):26. doi: 10.3390/molecules26010026 (PMC7793138; doi:10.3390/molecules26010026)
Supplement: Supplementary file 1 [file molecules-26-00026-s001.pdf]

Supporting Information for Publication

Crystalline peroxosolvates: nature of the coformer, hydrogen-bonded networks and clusters, intermolecular interactions

Alexander G. Medvedev,[†] Andrei V. Churakov,[†] Petr V. Prikhodchenko,[†] Ovadia Lev,[§,*] Mikhail V. Vener[†,‡,*]

[†]*Kurnakov Institute of General and Inorganic Chemistry, Russian Academy of Sciences, Leninskii prosp. 31, Moscow 119991, Russia*

[‡]*Department of Quantum Chemistry, Mendeleev University of Chemical Technology, Miusskaya Square 9, Moscow 125047, Russia*

[§]*The Casali Center of Applied Chemistry, The Institute of Chemistry, The Hebrew University of Jerusalem, Jerusalem 91904, Israel.*

Contents

Table S1 2

Table S2 17

References 27

Table S1. Structure features of peroxosolvates with localized protons.

	Coformer (Cof)	Refcode [a)]	Stoichiometry	X,Y [c)]	$n(H_2O_2)$ [d)]	N_1 [e)]	N_2 [f)]	Contact Me-O_p, Å	Ref
**Salts of inorganic and carboxylic acids**									
1	Ammonium oxalate, $(NH_4)_2C_2O_4$,	AMOXPH10	Cof•($0.94H_2O_2$•$0.06H_2O$) [b)]	O,O	1	2	2		[1]
2	Lithium oxalate, $Li_2C_2O_4$	LIOXPH	Cof•H_2O_2	O,O	1	2	0	(Li): 2.088; 2.284	[2]
3	Sodium oxalate, $Na_2C_2O_4$	NAOXAP	Cof•H_2O_2	O,O	1	2	0	(Na): 2.423; 2.435	[3]
		NAOXAP11= 246301[g)], NAOXAP01	Cof•H_2O_2	--//--	--//--	--//--	--//--	(Na): 2.388; 2.425	[4]
4	Potassium oxalate, $K_2C_2O_4$	KOXPHY11, (KOXPHY10= 246300)[g)] KOXPHY01	Cof•H_2O_2	O,O	1	2	0	(K): 2.856 - 3.240	[5,6]
5	Tripotassium tris(oxalato-κ^2*O*,*O′*)-aluminate 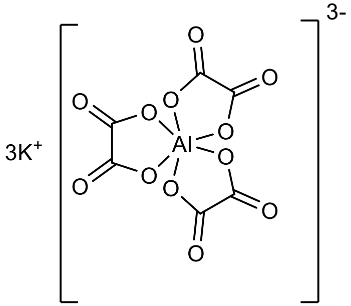	GADMUU, 170728	Cof•H_2O_2•($0.75H_2O_2$•$0.25H_2O$)	O,O	2	2; 2	1; 1	(K): 2.776 - 3.370	[7]
6	Guanidinium sulfate, $(CN_3H_6)_2SO_4$	BOHLOC	Cof•H_2O_2	O,O	1	2	1		[8]
7	Ammonium succinate, $(NH_4)_2C_4H_4O_4$	BAMXAR	Cof•$2H_2O_2$	O,O	1	2	0		[9]
8	Potassium hydrogen phthalate, 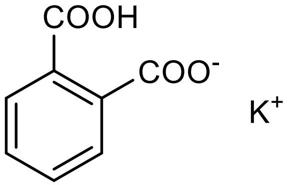	YUHTAW	Cof•$0.5H_2O_2$	O,O	1	2	0	(K): 2.798 - 3.482	[10]

	Coformer (Cof)	Refcode [a)]	Stoichiometry	X,Y[c)]	$n(H_2O_2)$[d)]	N_1[e)]	N_2[f)]	Contact Me-O_p, Å	Ref
9	Potassium fluoride, KF	22365, 22366	Cof•$2H_2O_2$	F,F	2	2; 2	0; 0	(K): 2.785-3.167	[11]
10	Rubidium fluoride, RbF	200383, 200384	Cof•H_2O_2	O,O	1	2	0	(Rb): 2.970-3.097	[12]
11	Ammonium fluoride, NH_4F	28552	Cof•H_2O_2	F,F	1	2	2		[13]
12	Nonasodium tetrasulfate chloride, $Na_9(SO_4)_4Cl$	171004, (1715)[g)]	Cof•$2H_2O_2$	O,O	4	2; 2; 2; 2	0; 0; 0; 0	(Na): 2.354 – 2.420	[14]
13	Nonasodium tetrasulfate bromide, $Na_9(SO_4)_4Br$	171005	Cof•H_2O_2	O,O	1	2	0	(Na): 2.379 – 2.391	[14]
14	Guanidinium oxalate, $(CN_3H_6)_2C_2O_4$	GADOXP10	Cof•H_2O_2•$2H_2O$	O,O	1	2	2		[15]
15	Sodium carbonate, Na_2CO_3	200044, WUTKUT08,(1304)[g)]	Cof•$1.5H_2O_2$	O,O	2	2; 2	0; 0		[16,17]
		281382,WUTKUT	--//--	--//--	--//--	--//--	--//--		[18]
		281383, WUTKUT01	--//--	--//--	--//--	--//--	--//--		
		281384, WUTLEE	--//--	--//--	--//--	--//--	--//--	(Na): 2.401 - 2.504	
		281385, WUTKUT02	--//--	--//--	--//--	--//--	--//--	(Na): 2.388 - 2.511	
		281386, WUTKUT03	--//--	--//--	--//--	--//--	--//--	(Na): 2.377 - 2.497	
		281387, WUTKUT04	--//--	--//--	--//--	--//--	--//--	(Na): 2.371 - 2.492	
		281388, WUTKUT05	--//--	--//--	--//--	--//--	--//--		
		281389, WUTKUT06	--//--	--//--	--//--	--//--	--//--		
		281390, WUTKUT07	--//--	--//--	--//--	--//--	--//--		
16	Ammonium carbonate, $(NH_4)_2CO_3$	262530, WUXSIT	Cof•H_2O_2	O,O	2	2; 2	0; 0		[19]
17	Caesium carbonate, Cs_2CO_3	262531, WUXSOZ	Cof•$3H_2O_2$	O,O	4	2; 2; 2; 2	0; 0; 0; 0	(Cs): 3.056 - 3.467	
18	Potassium hydrogendioxoperoxocarbonate, $K(H(O_2)CO_2)$	407779	Cof•H_2O_2	O,O	1	2	0	(K): 2.747- 2.820	[20]
19	Tetrapotassium bis(hydrogenphosphate), $K_4(HPO_4)_2$	418249	Cof•$3H_2O_2$	O,O	3	2; 2; 2	0; 0; 0	(K): 2.695 – 3.303	[21]
20	Pottasium dioxoperoxocarbonate, $K_2(O_2)CO_2$	421906	Cof•$3.5H_2O_2$	O,O	5	2; 2; 2; 2; 2	0; 0; 0; 0; 0	(K): 2.772 - 3.290	[22]

	Coformer (Cof)	Refcode [a)]	Stoichiometry	X,Y [c)]	$n(H_2O_2)$ [d)]	N_1 [e)]	N_2 [f)]	Contact Me-O_p, Å	Ref
21	Tetraphenylphosphonium chloride, 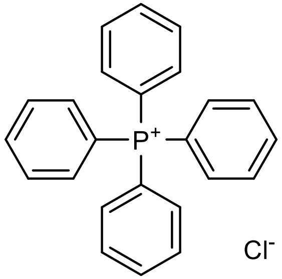	CAZHAN	Cof•H_2O_2•(0.85H_2O_2•0.15H_2O)	Cl,Cl	2	2; 2	0; 0		[23]
22		CAZHAN01	Cof•H_2O_2•(0.9H_2O_2•0.1H_2O)	--//--	--//--	--//--	--//--		
23		CAZHAN02	Cof•H_2O_2•(0.73H_2O_2•0.27H_2O)	--//--	--//--	--//--	--//--		
24	Tetraphenylphosphonium bromide 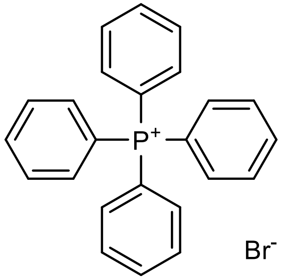	CAZHER [s7]	Cof•H_2O_2•(0.84H_2O_2•0.16H_2O)	Br,Br	2	2; 2	0; 0		
25		CAZHIV [s7]	Cof•H_2O_2•(0.5H_2O_2•0.5H_2O)	Br,Br	2	2; 2	0; 0		
26	Tetraphenylarsonium chloride 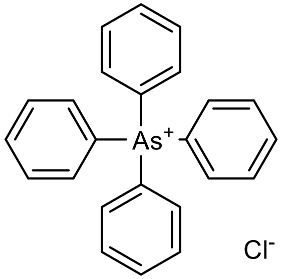	CAZHOB	Cof•H_2O_2•(0.85H_2O_2•0.15H_2O)	Cl,Cl	2	2; 2	0; 0		
27		CAZHUH	Cof•1.5H_2O_2	Cl,Cl	2	2; 2	0; 0		
28		CAZJAP	Cof•(0.98H_2O_2•0.02H_2O)	Cl,Cl	2	2; 2	0; 0		
29	Ammonium peroxotellurate, 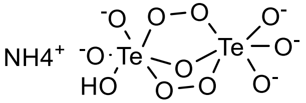	196444	Cof•(0.72H_2O_2•0.28H_2O)•H_2O	O,O	1	2	1		[24]

	Coformer (Cof)	Refcode [a)]	Stoichiometry	X,Y[c)]	n(H_2O_2)[d)]	N_1[e)]	N_2[f)]	Contact Me-O_p, Å	Ref
30	Caesium tetracyanoplatinate, $Cs_2(Pt(CN)_4)$	418911	Cof•H_2O_2	N,N	1	2	0	(Cs): 3.115 - 3.180	[25]
31	Rubidium tetracyanoplatinate, $Rb_2(Pt(CN)_4)$	418912	Cof•H_2O_2	N,N	1	2	0	(Rb): 2.980 -3.000	
32	Potassium tetracyanoplatinate, $K_2(Pt(CN)_4)$	418913	Cof•H_2O_2	N,N	1	2	0	(K): 2.843 - 2.918	
33	2,2'-Bipyridinium (2,2'-bipyridine)-oxo-diperoxo-vanadium 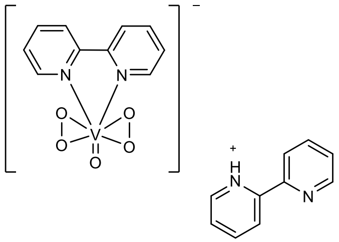	CEYXUZ	Cof•3.4H_2O_2•1.6H_2O	O,O	3	2; 2; 2	0; 2; 2		[26]
34	Tetra-n-butylammonium ((hydrogen peroxide)-(N,N,N-tris(2-((4-methylbenzene-1-sulfonyl)amino)ethyl)amine)-zinc aqua-(N,N,N-tris(2-((4-methylbenzene-1-sulfonyl)amino)ethyl)amine)-zinc) 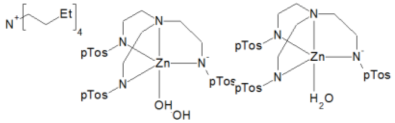	BABGUJ	Cof•(0.52H_2O_2•0.48H_2O)	O,O	1	2	0	(Zn): 2.172	[27]
35	Ammonium cyclopentazolate, 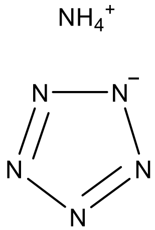	ZUWCIG	Cof•0.5H_2O_2	N,N	1	2	2		[28]
		ZUWCIG01	Cof•0.5H_2O_2	N,N	1	2	2		

	Coformer (Cof)	Refcode [a)]	Stoichiometry	X,Y[c)]	n(H_2O_2)[d)]	N_1[e)]	N_2[f)]	Contact Me-O_p, Å	Ref
**Molecular organic compounds with a lone electron pair on nitrogen and/or oxygen atoms**									
36	Lidocaine N-oxide 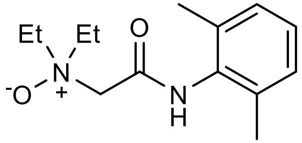	KELXEH	Cof•3H_2O_2	O,O	4	2; 2; 2; 2	0; 0; 2; 2		[29]
37	bis(dicyclohexylphosphino)ethane dioxide 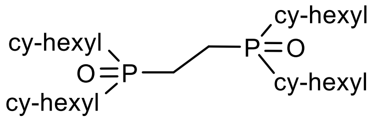	TOYTEJ	Cof•H_2O_2	O,O	1	2	0		[30]
38	tris(4-methylphenyl)(oxo)-phosphine 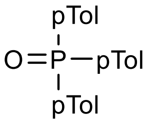	POMQEQ	Cof•H_2O_2	O,O	1	2	0		[31]
39	tris(2-methylphenyl)(oxo)-phosphine 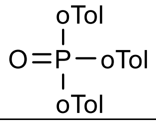	POMQIU	Cof•H_2O_2	O,O	1	2	0		

	Coformer (Cof)	Refcode [a)]	Stoichiometry	X,Y[c)]	n(H_2O_2)[d)]	N_1[e)]	N_2[f)]	Contact Me-O_p, Å	Ref
40	bis(2-methylphenyl)(oxo)phenyl-phosphine 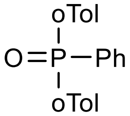	POMQOA	Cof•H_2O_2	O,O	1	2	0		
41	bis(tris(4-methylphenyl)(oxo)-phosphine) 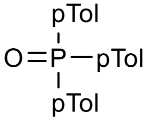	POMQUG	Cof•0.5H_2O_2	O,O	1	2	0		
42	(2-methylphenyl)(oxo)diphenyl-phosphine 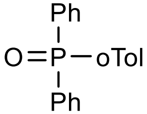	POMRAN	Cof•0.5H_2O_2	O,O	1	2	0		
43	Tri-t-butylphosphine oxide 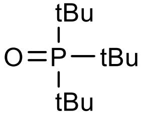	BAFGOH	Cof•H_2O_2	O,O	1	2	0		[32]
44	Triphenylphosphine oxide 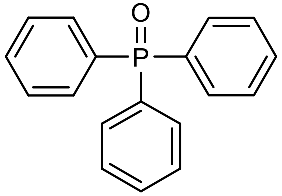	BAFJUQ, (TPPOPH)[g)]	Cof•1.5H_2O_2	O,O	2	2; 2	0; 1		[32,33]

	Coformer (Cof)	Refcode [a)]	Stoichiometry	X,Y [c)]	$n(H_2O_2)$ [d)]	N_1 [e)]	N_2 [f)]	Contact Me-O_p, Å	Ref
45	Tricyclohexylphosphine oxide 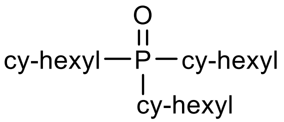	VANVOX	Cof•H_2O_2	O,O	1	2	0		[34]
46	3,6-Di-t-butyl-1,8-dioxo-1,8-diphospha-1,1,8,8-tetra-i-propyl-4,5-diazaocta-3,5-diene 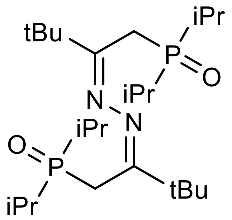	XETSUK	Cof•2H_2O_2	O,O	2	2; 2	0; 0		[35]
47	5,5'-dinitro-2H,2'H-3,3'-bi-1,2,4-triazole 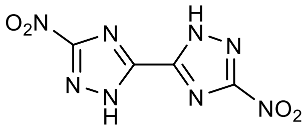	TUBNUC	Cof•0.4H_2O_2•0.2H_2O	N,N	1	2	2		[36]
48		TUBPOY	Cof•0.5H_2O_2	N,N	1	2	2		
49	Risperidone N-oxide 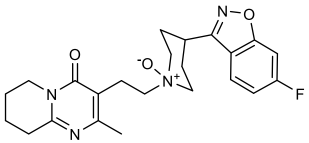	DATHIQ	Cof•H_2O_2•MeOH	O,O	1	2	0		[37]
50	1,2-dimethylimidazole 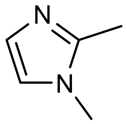	DOJMIZ	Cof•0.5H_2O_2	N,N	1	2	0		[38]

	Coformer (Cof)	Refcode [a)]	Stoichiometry	X,Y[c)]	$n(H_2O_2)$[d)]	N_1[e)]	N_2[f)]	Contact Me-O_p, Å	Ref
51	1,4-Diazabicyclo(2.2.2)octane N,N'-dioxide 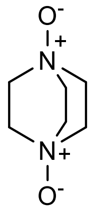	FURFIH	Cof•$3H_2O_2$	O,O	2	2; 2	0; 0		[39]
52	(Z)-N-benzylidene-1-phenylmethanamine oxide 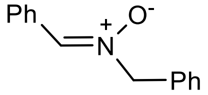	JELQOJ	Cof•H_2O_2	O,O	1	2	0		[40]
53	2,2′-Disulfanediylbis(pyridine N-oxide) 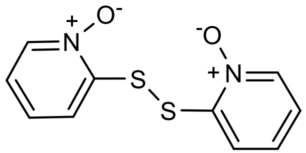	JESXEN	Cof•H_2O_2	O,O	1	2	0		[41]
54	Caffeine 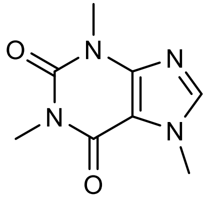	KUMRER	Cof•$0.5H_2O_2$	N,N	1	2	0		[h)]
55	(3aS,6R,7aS)-2-Acetyl-3,3,6-trimethyloctahydro-indazole 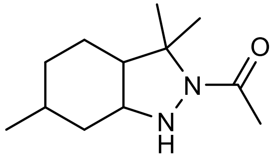	MUXHIX	Cof•H_2O_2	N,O	1	2	0		[42]

	Coformer (Cof)	Refcode [a)]	Stoichiometry	X,Y[c)]	$n(H_2O_2)$[d)]	N_1[e)]	N_2[f)]	Contact Me-O_p, Å	Ref
56	1,4-diazabicyclo-(2.2.2)-octane 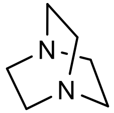	QOHXUH	Cof•$2H_2O_2$	N,O	1	2	1		[43]
57	2-(2-(Dimethylamino)-ethylmethylamino)-1,3,5-trimethyl-1,3,5-triaza-2-oxo-2λ^5-phosphorinane-4,6-dione 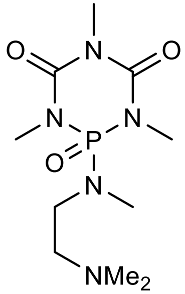	RIKJAW	Cof•H_2O_2	N,O	1	2	0		[44]
58	2,3,5,6-tetrakis(pyridin-2-yl)pyrazine 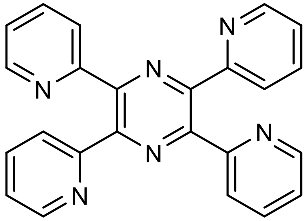	SEMXIU	Cof•$4.75H_2O_2$	O,O	6	2; 2; 2; 2; 2; 2	0; 0; 0; 1; 2; 2		[45]
59	2,6-bis-(Diphenyl-phosphoryl)-pyridine 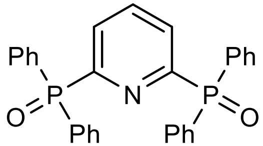	UKEFEV	Cof•H_2O_2	O,O	1	2	0		[46]

	Coformer (Cof)	Refcode [a)]	Stoichiometry	X,Y [c)]	n(H_2O_2) [d)]	N_1 [e)]	N_2 [f)]	Contact Me-O_p, Å	Ref
60	Urea, 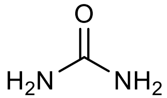	UREXPO11, 26444 (UREXPO) [g)] UREXPO01	Cof•H_2O_2	O,O	1	2	4		[47,48]
61	Bis(cyclohexyl)amine 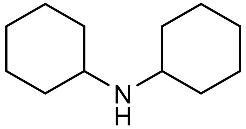	VAYGUY	Cof•0.5H_2O_2	N,N	1	2	0		h)
		VAYGUY01	--//--	--//--	--//--	--//--	--//--		[49]
62	Adenine 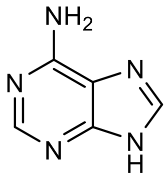	JOZZED	Cof•H_2O_2	N,N	1	2	2		[50]
63	Thymine 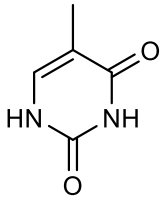	WINSAO	Cof•(0.55H_2O_2•0.45H_2O)	O,O	1	2	1		[51]
64		WINSAO01	Cof•(0.78H_2O_2•0.22H_2O)	--//--	--//--	--//--	--//--	--//--	[52]
65		YAFGEU	Cof•H_2O_2	--//--	--//--	--//--	--//--		
66	Melamine 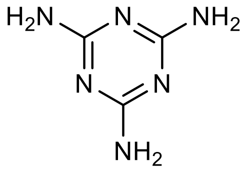	YAFFUJ	Cof•H_2O_2	N,N	1	2	4		

	Coformer (Cof)	Refcode [a)]	Stoichiometry	X,Y[c)]	$n(H_2O_2)$[d)]	N_1[e)]	N_2[f)]	Contact Me-O_p, Å	Ref
67	1-{2-(2,4-dichlorophenyl)-2-[(2,4-dichlorophenyl)methoxy]ethyl}-1H-imidazole 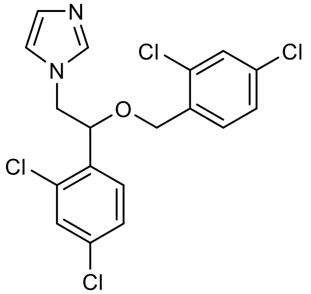	UDUROD	Cof•0.5H_2O_2	N,N	1	2	0		[53]
68	2-aminobenzimidazole 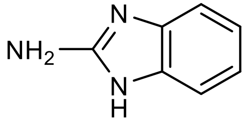	OHIJEX	2Cof•H_2O_2	N,N	1	2	4		[54]
69	2,4,6,8,10,12-hexanitro-2,4,6,8,10,12-hexaazaisowurtzitane 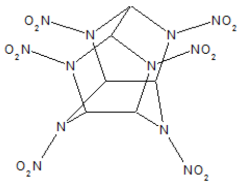	AZACIP, AZAMIZ, AZAMIZ01	Cof•0.5H_2O_2	O,O	1	2	0		[55]
**Amino acids, peptides and related zwitterions**									
70	N,N-dimethylglycine 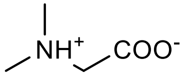	BONGES	Cof•H_2O_2	O,O	1	2	0		[56]

	Coformer (Cof)	Refcode [a)]	Stoichiometry	X,Y[c)]	$n(H_2O_2)$[d)]	N_1[e)]	N_2[f)]	Contact Me-O_p, Å	Ref
71	N,N,N-hydroxy-dimethylglycine 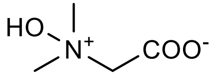	BONGIW	Cof•H_2O_2	O,O	1	2	0		
72	N,N,N-trimethylglycine (betaine) 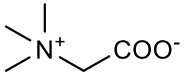	MEXTIU	Cof•($0.76H_2O_2$•$0.24H_2O$)	O,O	1	2	0	100 K	[57]
		MEXTIU01	Cof•($0.77H_2O_2$•$0.23H_2O$)	--//--	--//--	--//--	--//--	295 K	
73	L-serine 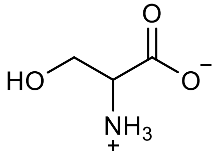	KULMOU, KULMOU01	Cof•H_2O_2	O,O	1	2	1		[58,59]
74		UDUWEX	Cof•($0.91H_2O_2$•$0.09H_2O$)	--//--	--//--	--//--	--//--		[h)]
75	Glycine 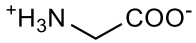	KULMUA, KULMUA01	Cof•$1.5H_2O_2$	O,O	3	2; 2; 2	1; 2; 2		[58,59]
76	2-aminobutyric acid 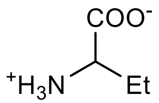	TANCAO	Cof•$1.5H_2O_2$	O,O	3	2; 2; 2	1; 1; 2		[59]
77	L-isoleucine 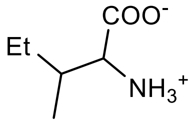	TANCES	Cof•H_2O_2•$0.5H_2O$	O,O	1	2	2		
78		TANDET	Cof•H_2O_2	O,O	1	2	1-bifur		
79	L-phenylalanine 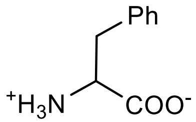	TANCIW	Cof•H_2O_2•$0.5H_2O$	O,O	1	2	2		
80		UDUWIB	Cof•H_2O_2•($0.43H_2O_2$•$0.07H_2O$)	O,O	2	2; 2	0; 2		[h)]
81	L-threonine 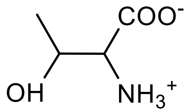	TANCOC	Cof•H_2O_2	O,O	1	2	3		[59]

	Coformer (Cof)	Refcode [a)]	Stoichiometry	X,Y [c)]	$n(H_2O_2)$ [d)]	N_1 [e)]	N_2 [f)]	Contact Me-O_p, Å	Ref
82	L-tyrosine 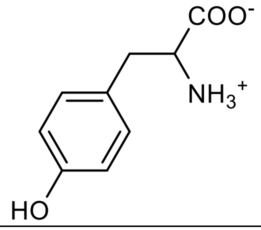	TANCUI	Cof•$2H_2O_2$	O,O	2	2; 2	0; 1		
83	β-alanine 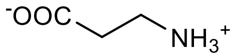	TANDAP	Cof•$2H_2O_2$	O,O	2	2; 2	0; 2		
84	L-tert-Leucine 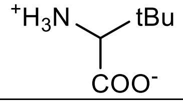	OJOCOH	Cof•H_2O_2•$0.5H_2O$	O,O	1	2	2		[h)]
85	Phenylserine 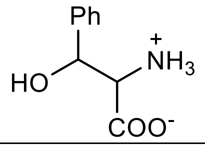	VILGAB	Cof•H_2O_2	O,O	1	2	2	H_2O_2 chains	[60]
86	Sarcosine 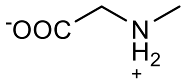	VILFUU	Cof•H_2O_2	O,O	3	2; 2; 2	1; 1; 1		
87	Pyridine-2-carboxylic acid 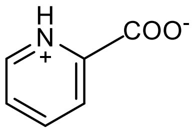	ANINES	Cof•H_2O_2	O,O	1	2	1		[61]
88	Pyridine-3-carboxylic acid 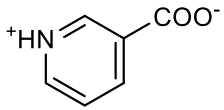	ANINAO	Cof•H_2O_2	O,O	1	2	0		

	Coformer (Cof)	Refcode [a)]	Stoichiometry	X,Y[c)]	$n(H_2O_2)$[d)]	N_1[e)]	N_2[f)]	Contact Me-O_p, Å	Ref
89	Pyridine-4-carboxylic acid 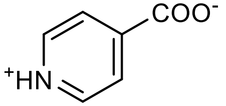	ANIMUH	Cof•$2H_2O_2$	O,O	2	2; 2	0; 1		
90	Cyclo-diglycine, 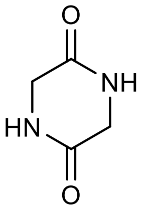	HOQRUD	Cof•$1.786H_2O_2$•$0.214H_2O$	O,O	1	2	1-bifur		[62]
91		HOQSAK	Cof•$2H_2O_2$	O,O	1	2	1-bifur		
92	Cyclo-disarcosine, 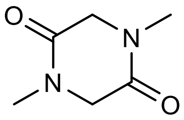	HOQSEO	Cof•H_2O_2	O,O	1	2	0		
93	Cyclo-dialanine, 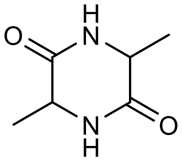	HOQSIS	Cof•$2H_2O_2$	O,O	2	2; 2	1; 1		
94	2- aminonicotinic acid, 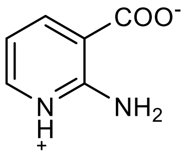	KELXAD	Cof•$1.5H_2O_2$	O,O	6	2; 2; 2; 2; 2; 2	1; 1; 1; 1; 1-bifur; 2		[29]

[a)] Alphanumeric refcodes corresponds to Cambridge Structural Database (CSD); numeric refcodes corresponds to Inorganic Crystal Structure Database (ICSD).

[b] Parenthesis denotes one crystallographical position occupied by both H_2O_2 and H_2O.

[c]) X and Y are atoms with electron lone pair in X···HOOH···Y fragment.

[d] Number of crystallographically independent H2O2 molecules.

[e] Number of H-bonds formed by H_2O_2 molecule as proton donor. If there are two or more crystallographically independent H_2O_2 molecules, than values are separated by commas.

[f] Number of H-bonds formed by H_2O_2 molecule as proton acceptor. If there are two or more crystallographically independent H_2O_2 molecules, than values are separated by commas.

[g] This structure has duplicate without localized protons.

[h] CSD Communication (Private communication).

Table S2. Refcodes of proton disordered peroxosolvates.

	Coformer (Cof)	Refcode [a)]	Stoichiometry	Comments	Ref
**Salts of inorganic and carboxylic acids**					
1	Potassium bis(oxalato)peroxo-vanadate(V), 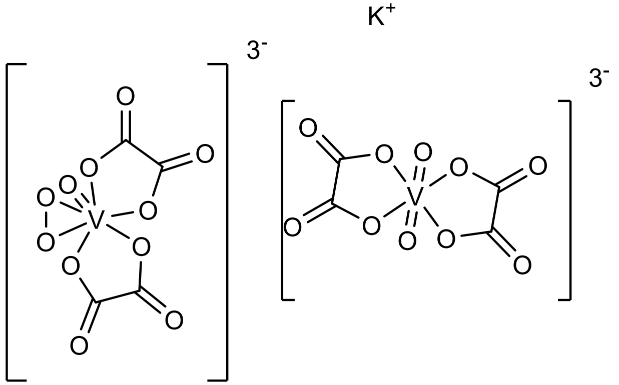	FAPJEL, 109697	Cof•($0.1H_2O_2$•$0.4H_2O$)	Not localized [b)]	[63]
2	Guanidinium diphosphate, 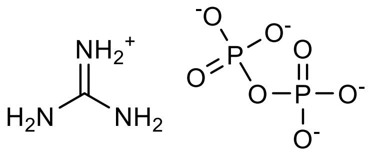	GUDPYQ, 2247	Cof•H_2O_2•$1.5H_2O$	Not localized	[64]
3	Guanidinium pyromellitate,	GUPMEL	Cof•$3H_2O_2$	Not localized	[65]
4	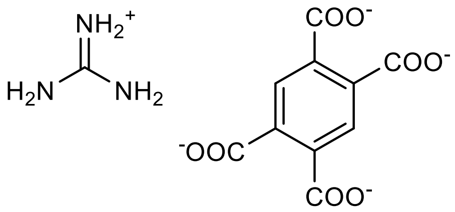	GUPYML	Cof•H_2O_2•$3H_2O$	Not localized	[66]

	Coformer (Cof)	Refcode [a)]	Stoichiometry	Comments	Ref
5	Hexarubidium (μ_2-peroxo)-tetrakis(oxalato-O,O')-tetraoxo-diuranium(VI), 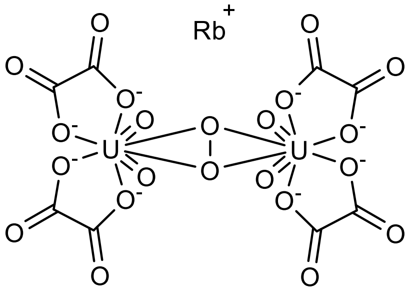	IGIHUD	Cof•3H_2O_2•4H_2O	Not localized	[67]
6	Hexacaesium) (μ_2-peroxo)-tetrakis(oxalato-O,O')-tetraoxo-diuranium(VI), 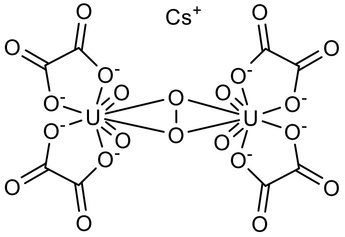	IGIJAL	Cof•3H_2O_2•4H_2O	Not localized	
7	Potassium triperoxo-(o-phenanthroline)-niobium(potassium triperoxo-(o-phenanthroline)-niobium(V) 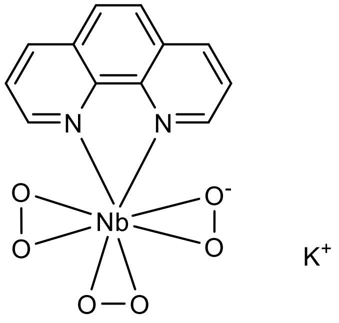	OPHENB10	Cof•H_2O_2•3H_2O	Not localized	[68]

	Coformer (Cof)	Refcode [a)]	Stoichiometry	Comments	Ref
8	Rubidium oxalate, $Rb_2C_2O_4$	RBOXPH10	Cof•H_2O_2	Not localized	[6]
9	Dirubidium dodecahydroxo-closododecaborate, $Rb_2(B_{12}(OH)_{12})$	181873	Cof•2H_2O_2	Short Op-Op distance (1.259 Å)	[69]
10	Trisodium tetraperoxovanadate, $Na_3(V(O_2)_4)$	81292	Cof•H_2O_2•10.5H_2O	Not localized	[70]
11	Dibarium silicododecatungstate, $Ba_2(SiW_{12}O_{40})$	161719	Cof•4H_2O_2•11H_2O	Not localized	[71]
12	Hexapotassium dodecafluorotriperoxotrizirconate, $K_6(Zr_3F_{12}(O_2)_3)$	80814	Cof•2H_2O_2•H_2O	Not localized	[72]
13	Potassium octafluorodiperoxodititanate, $K_4(Ti_2F_8(O_2)_2)$	68668	Cof•H_2O_2	Not localized	[73]
14	Strontium peroxide, α-SrO_2	24764	Cof•2H_2O_2	Not localized	[74]
15	Strontium peroxide, β-SrO_2	24765	Cof•2H_2O_2	Not localized	
16	Barium peroxide, α-BaO_2	24776	Cof•2H_2O_2	Not localized	[75]
17	Barium peroxide, β-BaO_2	24777	Cof•2H_2O_2	Not localized	
18	Barium peroxide, γ-BaO_2	24778	Cof•2H_2O_2	Not localized	
19	Barium peroxide, BaO_2	24779	Cof•H_2O_2	Not localized	[76]
20		44406	Cof•H_2O_2•2H_2O	Not localized	[75]
21	tris(Guanidinium) (ethylenediaminetetra-acetato N,N'-dioxide)-bis(peroxo)-tantalum(V) hydrogenperoxide solvate hydrate, 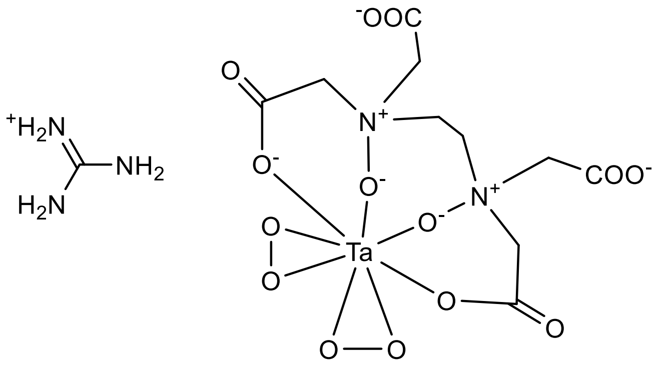	ABUNUH	Cof•(0.68H_2O_2•0.32H_2O) •2H_2O	Not localized	[77]

	Coformer (Cof)	Refcode [a)]	Stoichiometry	Comments	Ref
22	Tetrabutylammonium (hydrogen peroxo)-(peroxo)-oxo-(1,10-phenanthroline)-vanadium bis(peroxo)-(oxo)-(1,10-phenanthroline)-vanadium, 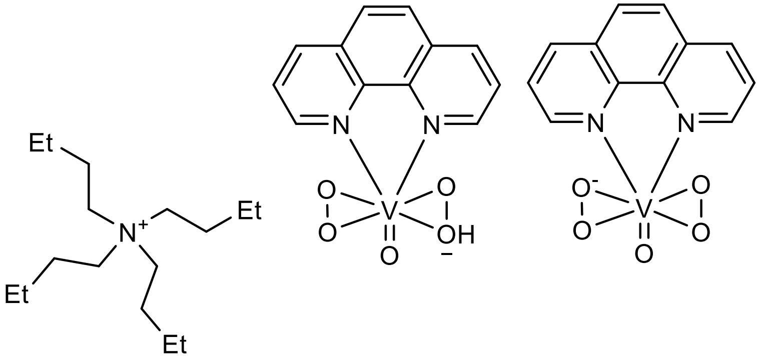	CELNIS	Cof•3H_2O_2•H_2O	Short Op-Op distance (1.340 Å, 1.382 Å)	[78]
23	tris(Guanadinium) (N,N'-dioxido-ethylenediaminetetraacetato)-bis(peroxo)-niobium(V), 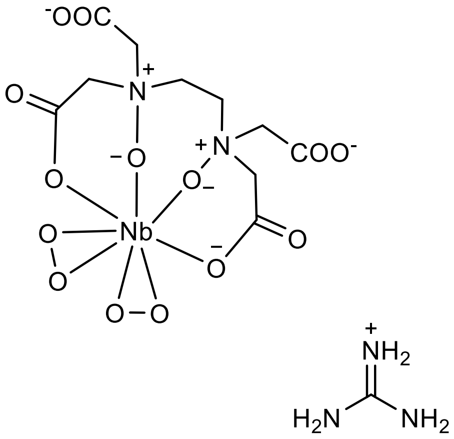	XUVRIP	Cof•H_2O_2•3H_2O	Not localized	[79]

	Coformer (Cof)	Refcode a)	Stoichiometry	Comments	Ref
24	pentakis(Guanidinium)(μ_2-tartrato)-(μ_2-hydrogen tartrato)-tetrakis(peroxo)-di-niobium(V), 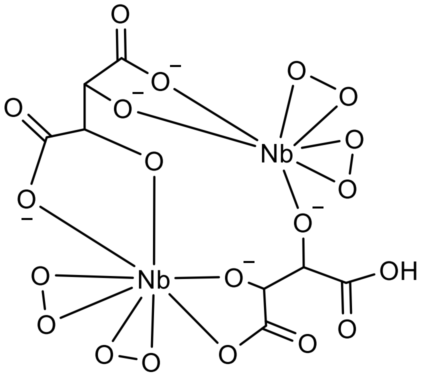 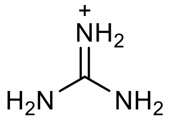	RASBAP	Cof•H_2O_2•6H_2O	Not localized	[80]
25	hexakis(Triphenyl(methyl)phosphonium) triiodo-tribromo-bismuth tris(μ_2-iodo)-triiodo-tribromo-di-bismuth, 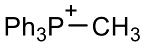 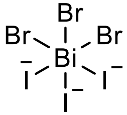 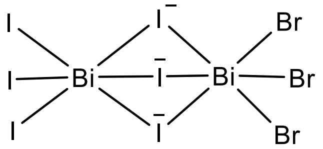	NAJMES	Cof•H_2O_2	Peroxide H atom positions determined incorrectly.	[81]
26	tris(Tetra-n-butylammonium) (m12-arsenato)-tetracosakis(m2-oxo)-dodecaoxo-dodeca-molybdenum, $(C_{16}H_{36}N)_3[AsMo_{12}O_{40}]$	LUGSIP	Cof•0.5H_2O_2	Short Op-Op distance (1.292 Å). Peroxide H atom positions determined incorrectly.	[82]

	Coformer (Cof)	Refcode a)	Stoichiometry	Comments	Ref
27	((2-(bis(Pyridin-2-ylmethyl)amino)ethyl)(pyridinium-2-ylmethyl)amino)acetato)-(peroxo-O,O')-chromium(IV) diperchlorate, 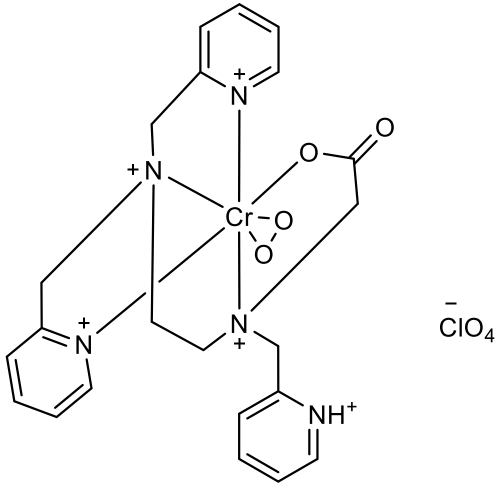	LUCJEA	Cof•$3H_2O_2$•H_2O	Angle Op-Op-H=75.5°	[83]
28	2,2'-Bipyridinium bis((2,2'-bipyridine)-oxo-diperoxo-vanadium), 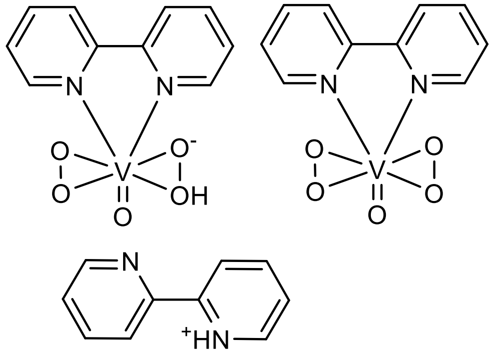	CEYXOT	Cof•$0.5H_2O_2$•$5.5H_2O$	Not localized	[84]

	Coformer (Cof)	Refcode [a)]	Stoichiometry	Comments	Ref
29	Tripotassium (oxalato-O,O')-oxo-bis(peroxo)-vanadium perhydrate 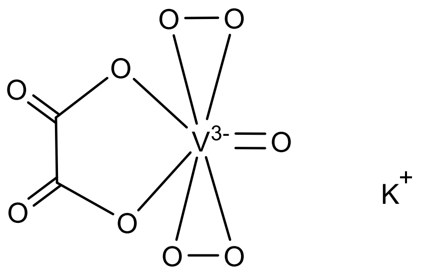	CIBBOE	Cof•$2H_2O_2$	Not localized	[85]
30	Tetra-ammonium (μ_2-(R)-3-phenyllactato-O,O,O')-(μ2-(S)-3-phenyllactato-O,O,O')-bis(oxo-(peroxo-O,O')-vanadium(V)) methanol solvate bis(hydrogen peroxide) dihydrate 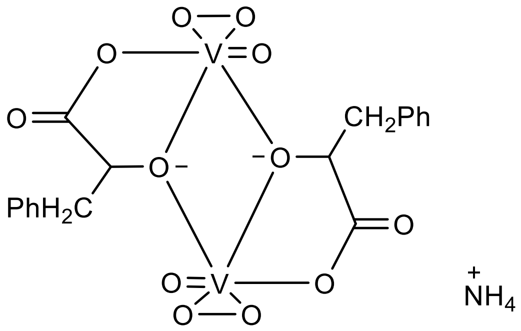	YATTIX	Cof•$2H_2O_2$•$2CH_3OH$•$2H_2O$	Not localized	[86]
31	cis,trans-Dichloro-dihydroxy-bis(isopropylamine)-platinum(IV), 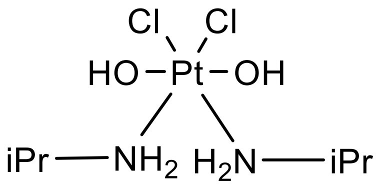	CAXCAF	Cof•$0.5H_2O_2$	Not localized	[87]
32	Trans-diammine-trans-dichloro-trans-dihydroxoplatinum(IV), $PtCl_2(NH_3)_2(OH)_2$	49991	Cof•H_2O_2	Not localized	[88]

	Coformer (Cof)	Refcode [a)]	Stoichiometry	Comments	Ref
**Molecular organic compounds with a lone electron pair on nitrogen and/or oxygen atoms**					
33	Tris(2-(oxy-N,N-dimethylammoniomethyl)phenyl)phosphine oxide, 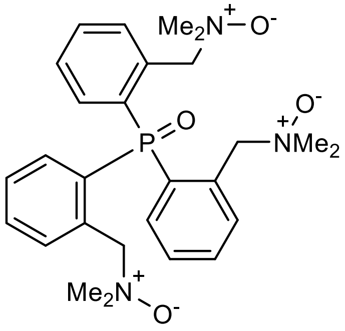	EKULUR	Cof•4H_2O_2•1.5H_2O	Not localized	[89]
34	Hexamethylenetetramine-oxide 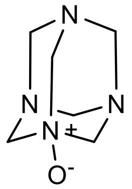	HMTOXH	Cof•H_2O_2•H_2O	Not localized	[90]
35	2,4,6-triamino-5-nitropyrimidine-1,3-dioxide, 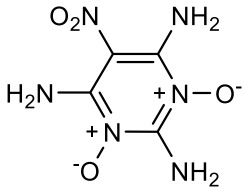	HOJCAN	Cof•0.5H_2O_2	Not localized	[91]

	Coformer (Cof)	Refcode [a)]	Stoichiometry	Comments	Ref
36	8,8'-(dihydroxy-tellanediyl)bis(N,N-dimethylnaphthalen-1-amine), 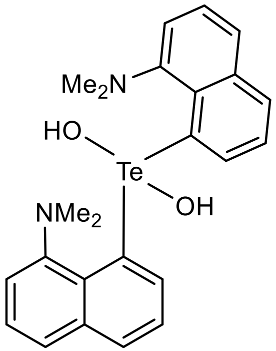	MAQRIH	Cof•H_2O_2•H_2O	Not localized	[92]
37	1-Phenyl-4-(4-phthalimidobutyl)piperazine-1,4-dioxide, 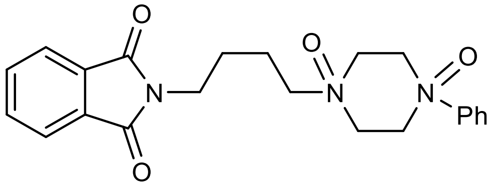	ROLPAJ	Cof•H_2O_2•(0.6H_2O_2•0.4 H_2O)	Not localized	[93]
38	bis((1-(2-Pyridylazo)-2-naphtholato-O,N,N')-(μ_2-oxo)-oxo-vanadium(V)), 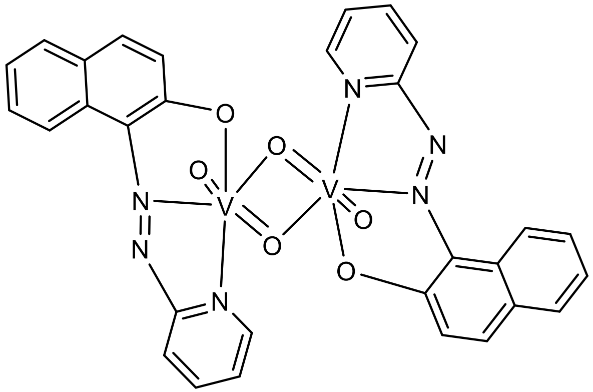	VAYMAJ	Cof•H_2O_2	Not localized	[94]

	Coformer (Cof)	Refcode [a)]	Stoichiometry	Comments	Ref
39	2-Diethylamino-1,3,5-trimethyl-1,3,5-triaza-2-oxo-2λ^5-phosphinane-4,6-dione, 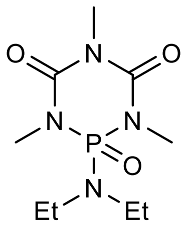	YECTOP	Cof•0.5H_2O_2	Not localized	[95]
**Others**					
40	Water, H_2O	24073	2Cof•H_2O_2	Not localized	[96]

[a)] Alphanumeric refcodes corresponds to Cambridge Structural Database (CSD); numeric refcodes corresponds to Inorganic Crystal Structure Database (ICSD).

[b)] Peroxide H atom positions were not determined.

References

1. Pedersen, B.F. The crystal structure of ammonium oxalate monoperhydrate. *Acta Crystallogr., Sect. B* **1972**, *28*, 746–754, doi:10.1107/S0567740872003139.

2. Pedersen, B.F.; Larsen, T.K.; Soling, H.; Torbjörnsson, L.; Werner, P.-E.; Junggren, U.; Lamm, B.; Samuelsson, B. The Crystal Structure of Lithium Oxalate Monoperhydrate, $Li_2C_2O_4{\cdot}H_2O_2$. *Acta Chem. Scand.* **1969**, *23*, 1871–1877, doi:10.3891/acta.chem.scand.23-1871.

3. Pedersen, B.F.; Pedersen, B.; Ledaal, T.; Seip, H.M. The Crystal Structure of Sodium Oxalate Perhydrate $Na_2C_2O_4{\cdot}H_2O_2$. *Acta Chem. Scand.* **1964**, *18*, 1454–1468, doi:10.3891/acta.chem.scand.18-1454.

4. Pedersen, B.F.; Kvick, Å.;. Neutron diffraction study of sodium oxalate monoperhydrate at 123 K. *Acta Crystallogr. Sect. C Cryst. Struct. Commun.* **1989**, *45*, 1724–1727, doi:10.1107/S0108270189003318.

5. Pedersen, B.F.; Kvick, Å.;. Neutron diffraction study of potassium oxalate monoperhydrate at 123 K. *Acta Crystallogr. Sect. C Cryst. Struct. Commun.* **1990**, *46*, 21–23, doi:10.1107/S010827018900332X.

6. Pedersen, B.F.; Seip, H.M.; Santesson, J.; Holmberg, P.; Eriksson, G.; Blinc, R.; Paušak, S.; Ehrenberg, L.; Dumanović, J. The Crystal Structure of Potassium and Rubidium Oxalate Monoperhydrates, $K_2C_2O_4{\cdot}H_2O_2$ and $Rb_2C_2O_4{\cdot}H_2O_2$. *Acta Chem. Scand.* **1967**, *21*, 779–790, doi:10.3891/acta.chem.scand.21-0779.

7. Chohan, S.; Pritchard, R.G. Tripotassium tris(oxalato-κ^2O,O′)aluminate bis(hydrogen peroxide) hydrate, the first example of a cyclic hydrogen-bonded H_2O_2 dimer. *Acta Crystallogr. Sect. C Cryst. Struct. Commun.* **2003**, *59*, m187–m189, doi:10.1107/S0108270103006905.

8. Churakov, A. V.; Medvedev, A.G.; Navasardyan, M.A.; Grishanov, D.A.; Prikhodchenko, P. V. The Crystal Structure of Guanidinium Sulphate Hemiperoxosolvate. *Propellants, Explos. Pyrotech.* **2018**, *43*, 859–861, doi:10.1002/prep.201800177.

9. Churakov, A. V.; Medvedev, A.G.; Mikhailov, A.A.; Tripol'skaya, T.A.; Prikhodchenko, P. V. Crystal structure of ammonium succinate peroxosolvate. *J. Struct. Chem.* **2014**, *55*, 1390–1394, doi:10.1134/S0022476614080022.

10. Kariuki, B.M.; Jones, W. Potassium Hydrogen Phthalate Hemiperhydrate. *Acta Crystallogr. Sect. C Cryst. Struct. Commun.* **1995**, *51*, 1128–1130, doi:10.1107/S0108270194012497.

11. Sarin, V.A.; Dudarev, V.Y.; Dobrynina, T.A.; Fykin, L.E.; Zavodnik, V.E. X-ray and neutron-diffraction study of $KF{\cdot}2H_2O_2$. *Kristallografiya* **1976**, *21*, 929–936.

12. Sarin, V.A.; Dudarev, V.Y.; Dobrynina, T.A.; FYKIN, L.E.; Zavodnik, V.E. X-ray and neutron-diffraction study of $RbF{\cdot}2H_2O_2$ crystals. *Kristallografiya* **1977**, *22*, 982–987.

13. Sarin, V.A.; Dudarev, V.Y.; Dobrynina, T.A.; Zavodnik, V.E. X-ray structural investigation of $NH_4F·H_2O_2$ crystals. *kristallografiya* 1979, *24*, 824–825.

14. Pritchard, R.G.; Begum, Z.; Lau, Y.F.; Austin, J. Structures of $Na_9[SO_4]_4X·2H_2O_2$, where X = Cl or Br, in which the halide anions orchestrate extended orientation sequences of H_2O_2 solvate molecules. *Acta Crystallogr. Sect. B Struct. Sci.* **2005**, *61*, 663–668, doi:10.1107/S010876810503212X.

15. Adams, J.M.; Pritchard, R.G. The crystal structure of guanidinium oxalate dihydrate monoperhydrate. *Acta Crystallogr. Sect. B Struct. Crystallogr. Cryst. Chem.* **1976**, *32*, 2438–2440, doi:10.1107/S0567740876007929.

16. de C. T. Carrondo, M.A.A.F.; Griffith, W.P.; Jones, D.P.; Skapski, A.C. X-Ray crystal structure of the industrial bleaching agent 'sodium percarbonate'[sodium carbonate–hydrogen peroxide (2/3)]. *J. Chem. Soc., Dalt. Trans.* **1977**, 2323–2327, doi:10.1039/DT9770002323.

17. Adams, J.M.; Pritchard, R.G. The crystal structure of sodium percarbonate: an unusual layered solid. *Acta Crystallogr. Sect. B Struct. Crystallogr. Cryst. Chem.* **1977**, *33*, 3650–3653, doi:10.1107/S0567740877011790.

18. Pritchard, R.G.; Islam, E. Sodium percarbonate between 293 and 100 K. *Acta Crystallogr. Sect. B Struct. Sci.* **2003**, *59*, 596–605, doi:10.1107/S0108768103012291.

19. Medvedev, A.G.A.G.; Mikhaylov, A.A.A.A.; Churakov, A.V.A.V.; Prikhodchenko, P.V.P. V.; Lev, O. Ammonium and caesium carbonate peroxosolvates: Supramolecular networks formed by hydrogen bonds. *Acta Crystallogr. Sect. C Cryst. Struct. Commun.* **2012**, *68*, i20–i24, doi:10.1107/S0108270112006701.

20. Adam, A.; Mehta, M. $KH(O_2)CO_2·H_2O_2$—An Oxygen-Rich Salt of Monoperoxocarbonic Acid. *Angew. Chemie Int. Ed.* **1998**, *37*, 1387–1388, doi:10.1002/(SICI)1521-3773(19980605)37:10<1387::AID-ANIE1387>3.0.CO;2-3.

21. Oeckler, O.; Montbrun, L. Crystal Structures of Alkali Metal Peroxodiphosphates. *Zeitschrift für Anorg. und Allg. Chemie* **2008**, *634*, 279–287, doi:10.1002/zaac.200700390.

22. Hinrichs, F.; Adam, A. Ein neues Salz der Monoperoxokohlensäure: $K_2(O_2)CO_2·3.5H_2O_2$. *Zeitschrift für Anorg. und Allg. Chemie* **2011**, *637*, 426–429, doi:10.1002/zaac.201000322.

23. Churakov, A. V.; Prikhodchenko, P. V.; Howard, J.A.K. The preparation and crystal structures of novel perhydrates $Ph_4X^+Hal^-·nH_2O_2$: anionic hydrogen-bonded chains containing hydrogen peroxide. *CrystEngComm* **2005**, *7*, 664, doi:10.1039/b511834d.

24. Mikhaylov, A.A.; Medvedev, A.G.; Churakov, A. V.; Grishanov, D.A.; Prikhodchenko, P. V.; Lev, O. Peroxide Coordination of Tellurium in Aqueous Solutions. *Chem. A Eur. J.* **2016**, *22*, 2980–2986, doi:10.1002/chem.201503614.

25. Mühle, C.; Peters, E.-M.; Jansen, M. New Hydrogen Peroxide Adducts of Alkali Metal Tetracyanoplatinates $A_2[Pt(CN)_4]·H_2O_2$ (A = K, Rb, Cs).

Zeitschrift für Naturforsch. B **2009**, *64*, 111–115, doi:10.1515/znb-2009-0115.

26. Stomberg, R.; Szentivanyi, H.; Hämäläinen, R.; Kohl, F.X.; Seip, R. The Crystal Structure of 2,2'-Bipyridinium(1+) (2,2'-Bipyridine)oxodiperoxovanadate(1-)-(3+x)-hydrogen peroxide-(2-x)-water, $(C_{10}H_9N_2)[VO(O_2)_2(C_{10}H_8N_2)]\cdot(3+x)H_2O_2\cdot(2-x)H_2O$, x = 0.4, at -100°C. *Acta Chem. Scand.* **1984**, *38a*, 121–128, doi:10.3891/acta.chem.scand.38a-0121.
27. Wallen, C.M.; Bacsa, J.; Scarborough, C.C. Hydrogen Peroxide Complex of Zinc. *J. Am. Chem. Soc.* **2015**, *137*, 14606–14609, doi:10.1021/jacs.5b10450.
28. Luo, J.; Xia, H.; Zhang, W.; Song, S.; Zhang, Q. A promising hydrogen peroxide adduct of ammonium cyclopentazolate as a green propellant component. *J. Mater. Chem. A* **2020**, *8*, 12334–12338, doi:10.1039/D0TA03010D.
29. Grishanov, D.A.; Navasardyan, M.A.; Medvedev, A.G.; Lev, O.; Prikhodchenko, P. V.; Churakov, A. V. Hydrogen Peroxide Insular Dodecameric and Pentameric Clusters in Peroxosolvate Structures. *Angew. Chemie - Int. Ed.* **2017**, *56*, 15241–15245, doi:10.1002/anie.201709699.
30. Arp, F.F.; Ahn, S.H.; Bhuvanesh, N.; Blümel, J. Selective synthesis and stabilization of peroxides via phosphine oxides. *New J. Chem.* **2019**, *43*, 17174–17181, doi:10.1039/C9NJ04858H.
31. Arp, F.F.; Bhuvanesh, N.; Blümel, J. Hydrogen peroxide adducts of triarylphosphine oxides. *Dalt. Trans.* **2019**, *48*, 14312–14325, doi:10.1039/C9DT03070K.
32. Ahn, S.H.; Cluff, K.J.; Bhuvanesh, N.; Blümel, J. Hydrogen Peroxide and Di(hydroperoxy)propane Adducts of Phosphine Oxides as Stoichiometric and Soluble Oxidizing Agents. *Angew. Chemie Int. Ed.* **2015**, *54*, 13341–13345, doi:10.1002/anie.201505291.
33. Thierbach, D.; Huber, F.; Preut, H. Structure of triphenylphosphine oxide hemiperhydrate. *Acta Crystallogr. Sect. B Struct. Crystallogr. Cryst. Chem.* **1980**, *36*, 974–977, doi:10.1107/S0567740880005067.
34. Hilliard, C.R.; Bhuvanesh, N.; Gladysz, J.A.; Blümel, J. Synthesis, purification, and characterization of phosphine oxides and their hydrogen peroxide adducts. *Dalt. Trans.* **2012**, *41*, 1742–1754, doi:10.1039/C1DT11863C.
35. Čermák, J.; Kvíčalová, M.; Šabata, S.; Blechta, V.; Vojtíšek, P.; Podlaha, J.; Shaw, B.L. Diphosphinoazines (Z,Z)-$R_2PCH_2C(Bu^t)=NN=C(Bu^t)CH_2PR_2$ with R groups of various sizes and complexes {[(Z,Z)-$R_2PCH_2C(Bu^t)=NN=C(Bu^t)CH_2PR_2$]-[$\eta^3$-$CH_2C(CH_3)=CH_2PdCl]_2$}. *Inorganica Chim. Acta* **2001**, *313*, 77–86, doi:10.1016/S0020-1693(00)00376-5.
36. Wiscons, R.A.; Bellas, M.K.; Bennion, J.C.; Matzger, A.J. Detonation Performance of Ten Forms of 5,5′-Dinitro-2H,2H′-3,3′-bi-1,2,4-triazole (DNBT). *Cryst. Growth Des.* **2018**, *18*, 7701–7707, doi:10.1021/acs.cgd.8b01583.
37. Ravikumar, K.; Sridhar, B.; Manjunatha, S.G.; Thomas, S. Risperidone N -oxide hydrogen peroxide methanol solvate. *Acta Crystallogr. Sect. E*

Struct. Reports Online **2005**, *61*, o2515–o2517, doi:10.1107/S1600536805022002.

38. Laus, G.; Schwärzler, A.; Bentivoglio, G.; Hummel, M.; Kahlenberg, V.; Wurst, K.; Kristeva, E.; Schütz, J.; Kopacka, H.; Kreutz, C.; et al. Synthesis and Crystal Structures of 1-Alkoxy-3-alkylimidazolium Salts Including Ionic Liquids, 1-Alkylimidazole 3-oxides and 1-Alkylimidazole Perhydrates. *Zeitschrift für Naturforsch. B* **2008**, *63*, 447–464, doi:10.1515/znb-2008-0411.

39. Kay Hon, P.; Mak, T.C.W. Isolation and crystal structures of 1,3 molecular complexes of triethylenediamineN,N'-dioxide with hydrogen peroxide and water. *J. Crystallogr. Spectrosc. Res.* **1987**, *17*, 419–429, doi:10.1007/BF01180319.

40. Churakov, A. V.; Prikhodchenko, P. V.; Medvedev, A.G.; Mikhaylov, A.A. Crystal structure of (Z)- N -benzylidene-1-phenylmethanamine oxide hydrogen peroxide monosolvate. *Acta Crystallogr. Sect. E Crystallogr. Commun.* **2017**, *73*, 1666–1669, doi:10.1107/S2056989017014499.

41. Lynch, W.; Padgett, C.W. 2,2′-Disulfanediylbis(pyridine N -oxide)–hydrogen peroxide (1/1). *IUCrData* **2018**, *3*, x180320, doi:10.1107/S2414314618003206.

42. Jakob, F.; Herdtweck, E.; Bach, T. Synthesis and Properties of Chiral Pyrazolidines Derived from (+)-Pulegone. *Chem. - A Eur. J.* **2010**, *16*, 7537–7546, doi:10.1002/chem.201000219.

43. Laus, G.; Kahlenberg, V.; Wurst, K.; Lörting, T.; Schottenberger, H. Hydrogen bonding in the perhydrate and hydrates of 1,4-diazabicyclo[2.2.2]octane (DABCO). *CrystEngComm* **2008**, *10*, 1638, doi:10.1039/b807303a.

44. Neda, I.; Kaukorat, T.; Fischer, A.; Jones, P.G.; Schmutzler, R. Oxidationsreaktionen an 2-[2-(N,N-Dimethylamino)ethyl-methylamino]-1,3,5-trimethyl-1,3,5-triaza-2λ^3-phosphorinan-4,6-dion; hydrolyse und thermolyse eines perfluorpinakolylsubstituierten spirophosphorans. *J. Fluor. Chem.* **1994**, *69*, 35–40, doi:10.1016/0022-1139(93)03036-L.

45. Navasardyan, M.A.; Bezzubov, S.I.; Kuz'mina, L.G.; Prikhodchenko, P. V.; Churakov, A. V. Crystal structure of 2,3,5,6-tetrakis(pyridin-2-yl)pyrazine hydrogen peroxide 4.75-solvate. *Acta Crystallogr. Sect. E Crystallogr. Commun.* **2017**, *73*, 1793–1796, doi:10.1107/S2056989017015328.

46. Sevcik, R.; Necas, M.; Novosad, J. The synthesis and characterization of three oxidized derivatives of bis(diphenylphosphino)pyridine and their Sn(IV) complexes. *Polyhedron* **2003**, *22*, 1585–1593, doi:10.1016/S0277-5387(03)00291-2.

47. Fritchie Jnr, C.J.; McMullan, R.K. Neutron diffraction study of the 1:1 urea:hydrogen peroxide complex at 81 K. *Acta Crystallogr. Sect. B* **1981**, *B37*, 1086–1091, doi:10.1107/S0567740881005116.

48. Lu, C.-S.; Hughes, E.W.; Giguère, P.A. The Crystal Structure of the Urea—Hydrogen Peroxide Addition Compound CO(NH 2) 2 ·H 2 O 2. *J. Am. Chem. Soc.* **1941**, *63*, 1507–1513, doi:10.1021/ja01851a007.

49. Churakov, A. V.; Chetina, O. V.; Howard, J.A.K. Dicyclohexylamine hydrogen peroxide hemisolvate. *Acta Crystallogr. Sect. E Struct. Reports Online* **2006**, *62*, o3503–o3505, doi:10.1107/S1600536806028030.

50. Serra, M.A.; Dorner, B.K.; Silver, M.E. Structure of an adenine-hydrogen peroxide adduct. *Acta Crystallogr. Sect. C Cryst. Struct. Commun.* **1992**, *48*, 1957–1960, doi:10.1107/S0108270192002294.

51. Churakov, A. V; Howard, J.A.K. Thymine hydrogen peroxide 0.55-solvate 0.45-hydrate. *Acta Crystallogr., Sect. E* **2007**, *63*, o4483, doi:10.1107/S1600536807053585.

52. Chernyshov, I.Y.; Vener, M. V.; Prikhodchenko, P. V.; Medvedev, A.G.; Lev, O.; Churakov, A. V. Peroxosolvates: Formation Criteria, H_2O_2 Hydrogen Bonding, and Isomorphism with the Corresponding Hydrates. *Cryst. Growth Des.* **2017**, *17*, 214–220, doi:10.1021/acs.cgd.6b01449.

53. Kersten, K.M.; Breen, M.E.; Mapp, A.K.; Matzger, A.J. Pharmaceutical solvate formation for the incorporation of the antimicrobial agent hydrogen peroxide. *Chem. Commun.* **2018**, *54*, 9286–9289, doi:10.1039/C8CC04530E.

54. Churakov, A. V.; Grishanov, D.A.; Medvedev, A.G.; Mikhaylov, A.A.; Vener, M. V.; Navasardyan, M.A.; Tripol'skaya, T.A.; Lev, O.; Prikhodchenko, P. V. Stabilization of hydrogen peroxide by hydrogen bonding in the crystal structure of 2-aminobenzimidazole perhydrate. *CrystEngComm* **2020**, *22*, 2866–2872, doi:10.1039/D0CE00096E.

55. Bennion, J.C.; Chowdhury, N.; Kampf, J.W.; Matzger, A.J. Hydrogen Peroxide Solvates of 2,4,6,8,10,12-Hexanitro-2,4,6,8,10,12-hexaazaisowurtzitane. *Angew. Chemie Int. Ed.* **2016**, *55*, 13118–13121, doi:10.1002/anie.201607130.

56. Kapustin, E.A.; Minkov, V.S.; Boldyreva, E. V. Oxidative stress of H_2O_2 on N,N-dimethylglycine: formation of perhydrate crystals and more. *CrystEngComm* **2014**, *16*, 10165–10168, doi:10.1039/C4CE01835D.

57. Minkov, V.S.; Kapustin, E.A.; Boldyreva, E. V. Betaine 0.77-perhydrate 0.23-hydrate and common structural motifs in crystals of amino acid perhydrates. *Acta Crystallogr. Sect. C Cryst. Struct. Commun.* **2013**, *69*, 416–420, doi:10.1107/S0108270113005386.

58. Churakov, A. V; Prikhodchenko, P. V; Howard, J.A.K.; Lev, O. Glycine and l-serine crystalline perhydrates. *Chem. Commun.* **2009**, 4224, doi:10.1039/b906801e.

59. Prikhodchenko, P. V.; Medvedev, A.G.; Tripol'skaya, T.A.; Churakov, A. V.; Wolanov, Y.; Howard, J.A.K.K.; Lev, O. Crystal structures of natural amino acid perhydrates. *CrystEngComm* **2011**, *13*, 2399, doi:10.1039/c0ce00481b.

60. Navasardyan, M.A.; Grishanov, D.A.; Tripol'skaya, T.A.; Kuz'mina, L.G.; Prikhodchenko, P. V.; Churakov, A. V. Crystal structures of non-proteinogenic amino acid peroxosolvates: rare example of H-bonded hydrogen peroxide chains. *CrystEngComm* **2018**, *20*, 7413–7416, doi:10.1039/C8CE01486H.

61. Medvedev, A.G.; Mikhailov, A.A.; Prikhodchenko, P. V.; Tripol'skaya, T.A.; Lev, O.; Churakov, A. V. Crystal structures of

pyridinemonocarboxylic acid peroxosolvates. *Russ. Chem. Bull.* **2013**, *62*, 1871–1876, doi:10.1007/s11172-013-0269-9.

62. Churakov, A. V.; Grishanov, D.A.; Medvedev, A.G.; Mikhaylov, A.A.; Tripol'skaya, T.A.; Vener, M. V.; Navasardyan, M.A.; Lev, O.; Prikhodchenko, P. V. Cyclic dipeptide peroxosolvates: first direct evidence for hydrogen bonding between hydrogen peroxide and a peptide backbone. *CrystEngComm* **2019**, *21*, 4961–4968, doi:10.1039/C9CE00892F.

63. Stomberg, R.; Klemets, R.; Lundström, I.; Fontell, K.; Nielsen, C.J.; Urso, F.; Weidlein, J.; Zingaro, R.A. The Crystal Structures of Potassium Bis(oxalato)oxoperoxovanadate(V) Hemihydrate, $K_3[VO(O_2)(C_2O_4)_2]\cdot½H_2O$, and Potassium Bis(oxalato)dioxovanadate(V) Trihydrate, $K_3[VO_2(C_2O_4)_2]\cdot3H_2O$. *Acta Chem. Scand.* **1986**, *40a*, 168–176, doi:10.3891/acta.chem.scand.40a-0168.

64. Adams, J.M.; Ramdas, V. The crystal structure of guanidinium pyrophosphate monoperhydrate sesquihydrate. *Acta Crystallogr. Sect. B Struct. Crystallogr. Cryst. Chem.* **1978**, *34*, 2150–2156, doi:10.1107/S056774087800761X.

65. Adams, J.M.; Ramdas, V. The crystal structure of guanidinium pyromellitate triperhydrate. *Inorganica Chim. Acta* **1979**, *34*, L225–L227, doi:10.1016/S0020-1693(00)94643-7.

66. Adams, J.M.; Ramdas, V. The crystal structure of guanidinium pyromellitate trihydrate monoperhydrate. *Acta Crystallogr. Sect. B Struct. Crystallogr. Cryst. Chem.* **1978**, *34*, 2781–2785, doi:10.1107/S056774087800922X.

67. Qiu, J.; Vlaisavljevich, B.; Jouffret, L.; Nguyen, K.; Szymanowski, J.E.S.; Gagliardi, L.; Burns, P.C. Cation Templating and Electronic Structure Effects in Uranyl Cage Clusters Probed by the Isolation of Peroxide-Bridged Uranyl Dimers. *Inorg. Chem.* **2015**, *54*, 4445–4455, doi:10.1021/acs.inorgchem.5b00248.

68. Mathern, G.; Weiss, R. Structure des complexes peroxydiques des métaux de transition. II. Structure cristalline du triperoxo-(o-phénanthroline)niobate de potassium à trois molécules d'eau et de son perhydrate $KNb(O_2)_3(C_{12}H_8N_2)\cdot3H_2O$ et $KNb(O_2)_3(C_{12}H_8N_2)\cdot3H_2O\cdot H_2O_2$. *Acta Crystallogr. Sect. B Struct. Crystallogr. Cryst. Chem.* **1971**, *27*, 1582–1597, doi:10.1107/S0567740871004400.

69. Schölkopf, T.; Van, N.-D.; Schleid, T. $Rb_2[B_{12}(OH)_{12}]\cdot2H_2O$ and $Rb_2[B_{12}(OH)_{12}]\cdot2H_2O_2$: Hydrate and perhydrolate of rubidium dodecahydroxo-closo-dodecaborate. *Inorganica Chim. Acta* **2011**, *374*, 181–186, doi:10.1016/j.ica.2011.03.017.

70. Won, T.-J.; Barnes, C.L.; Schlemper, E.O.; Thompson, R.C. Two Crystal Structures Featuring the Tetraperoxovanadate(V) Anion and a Brief Reinvestigation of Peroxovanadate Equilibria in Neutral and Basic Solutions. *Inorg. Chem.* **1995**, *34*, 4499–4503, doi:10.1021/ic00121a031.

71. Churakov, A. V.; Legurova, E.A.; Dutov, A.A.; Prikhodchenko, P. V.; Tripol'skaya, T.A. Peroxide derivatives of heteropoly compounds with Keggin anions $[PW_{12}O_{40}]^{3-}$ and $[SiW_{12}O_{40}]^{4-}$: Synthesis and structure. *Russ. J. Inorg. Chem.* **2008**, *53*, 1187–1192, doi:10.1134/S0036023608080068.

72. Chernyshov, B.N.; Didenko, N.A.; Bukvetskii, B. V; Gerasimenko, A. V; Kavun, V.Y.; Sergienko, S.S. Synthesis and structure of $K_6Zr_{12}(O_2)_3 \cdot 2H_2O_2 \cdot H_2O$ polynuclear peroxofluorozirconates. *Russ.J. Inorg. Chem.* **1989**, *34*, 2786–2794.

73. Chernyshov, B.N.; Didenko, N.A.; Bukvetskii, B. V; Gerasimenko, A. V; Kavun, V.Y. Synthesis and structure of new peroxofluorotitanates. *Russ.J. Inorg. Chem.* **1989**, *34*, 2179–2186.

74. Vannerberg, N.G. On the system SrO_2-H_2O-H_2O_2. 1. The crystal structure of alpha-$SrO_2 \cdot 2H_2O_2$ and beta-$SrO_2 \cdot 2H_2O_2$. *Ark. KEMI* **1958**, *13*, 29–41.

75. Vannerberg, N.G. On the system BaO_2-H_2O-H_2O_2. 3. The crystal structure of alpha-BaO_2, beta-BaO_2, and gamma-$BaO_2 \cdot 2H_2O_2$ and $BaO_2 \cdot H_2O_2 \cdot 2H_2O$. *Ark. KEMI* **1959**, *14*, 125–145.

76. Vannerberg, N.G. On the system BaO_2-H_2O-H_2O_2. 1. Investigation of the existing phases and their preparation. 2. The structure of $BaO_2 \cdot H_2O_2$. *Ark. KEMI* **1959**, *14*, 147–160.

77. Bayot, D.; Tinant, B.; Devillers, M. Spectroscopic and Structural Characterizations of Novel Water-Soluble Tetraperoxo and Diperoxo[polyaminocarboxylato bis(N -oxido)]tantalate(V) Complexes. *Inorg. Chem.* **2004**, *43*, 5999–6005, doi:10.1021/ic049639k.

78. Šimuneková, M.; Šimunek, J.; Chrappová, J.; Schwendt, P.; Žák, Z.; Pavelčík, F. Dinucleating role of a strong hydrogen bond in crystal structure of $[N(C_4H_9)_4]\{[VO(HO_2)(O_2)(phen)][VO(O_2)_2(phen)]\} \cdot 3H_2O_2 \cdot H_2O$. *Inorg. Chem. Commun.* **2012**, *24*, 125–128, doi:10.1016/j.inoche.2012.08.003.

79. Bayot, D.; Tinant, B.; Mathieu, B.; Declercq, J.-P.; Devillers, M. Spectroscopic and Structural Characterizations of Novel Water-Soluble Peroxo[polyaminocarboxylato bis(N-oxido)]niobate(V) Complexes. *Eur. J. Inorg. Chem.* **2003**, *2003*, 737–743, doi:10.1002/ejic.200390102.

80. Bayot, D.; Tinant, B.; Devillers, M. Homo- and Heterobimetallic Niobium v and Tantalum v Peroxo-tartrate Complexes and Their Use as Molecular Precursors for Nb−Ta Mixed Oxides. *Inorg. Chem.* **2005**, *44*, 1554–1562, doi:10.1021/ic0484250.

81. Sharutin, V. V.; Yegorova, I. V.; Klepikov, N.N.; Boyarkina, E.A.; Sharutina, O.K. Synthesis and structure of bismuth complexes $[Ph_3MeP]^{6+}[BiI_3Br_3]^{3-}[Bi_2I_6Br_3]^{3-} \cdot H_2O_2$, $[Ph_3EtP]^{3+}[Bi_2I_9]^{3-}$,$[Ph_3MeP]^{3+}[Bi_3I_{12}]^{3-}$, $[Ph_3(iso\text{-}Pr)P]^{3+}[Bi_3I_{12}]^{3-} \cdot 2Me_2C{=}O$, and $[Ph_4Bi]^{3+}[Bi_5I_{18}]^{3-}$. *Russ. J. Inorg. Chem.* **2009**, *54*, 52–68, doi:10.1134/S0036023609010124.

82. Fidalgo, E.G.; Neels, A.; Stoeckli-Evans, H.; Süss-Fink, G. New iso and heteropolyoxomolybdates: synthesis and molecular structure of the anions $[Mo(VI)_8O_{26}(OH)]^{5-}$, $[Has(III)As(V)Mo(V)Mo(VI)_8O_{34}]^{6-}$ and $[HAs(III)As(V)Mo(V)Mo(VI)_8O_{34}\{Co(C_5H_5N)_2(H_2O)_3\}]^{4-}$. *Polyhedron* **2002**, *21*, 1921–1928, doi:10.1016/S0277-5387(02)01067-7.

83. de Sousa, D.P.; Bigelow, J.O.; Sundberg, J.; Que, L.; McKenzie, C.J. Caught! Crystal trapping of a side-on peroxo bound to $Cr_{(IV)}$. *Chem. Commun.* **2015**, *51*, 2802–2805, doi:10.1039/C4CC08785B.

84. Szentivanyi, H.; Stomberg, R.; Hämäläinen, R.; Kohl, F.X.; Seip, R. The Crystal Structure of 2,2’-Bipyridinium(1+) mu-Hydrogen-bis[(2,2’-bipyridine)oxodiperoxovanadate](1-)-x-hydrogen peroxide-(6-x)-water, (Hbipy)[H{VO(O_2)2bipy}$_2$]·xH_2O_2·(6-x)H_2O, x ~= 0.5, at -100 degrees C. *Acta Chem. Scand.* **1984**, *38a*, 101–107, doi:10.3891/acta.chem.scand.38a-0101.

85. Campbell, N.J.; Capparelli, M.V.; Griffith, W.P.; Skapski, A.C. On the existence of triperoxo vanadium complexes. X-ray crystal structures of $K_3[VO(O_2)_2(C_2O_4]\cdot H_2O_2$ and of $(NH_4)[VO(O_2)_2$(bipy)]·4H_2O. *Inorganica Chim. Acta* **1983**, *77*, L215–L216, doi:10.1016/S0020-1693(00)82620-1.

86. Schwendt, P.; Ahmed, M.; Marek, J. Complexation between vanadium (V) and phenyllactate: Synthesis, spectral studies and crystal structure of $(NEt_4)(NH_4)_3[V_2O_2(O_2)_2$(R-3-phlact)$_2$][$V_2O_2(O_2)$2(S-3-phlact)$_2$]·6$H_2O$, [3-phlact=3-phenyllactato(2−)]. *Inorganica Chim. Acta* **2005**, *358*, 3572–3580, doi:10.1016/j.ica.2005.06.039.

87. Barnard, C.F.J.; Hydes, P.C.; Griffiths, W.P.; Mills, O.S. A stable platinum complex perhydrate adduct: crystal stucture of cis,trans-[$PtCl_2(OH)_2$(2-NH_2Pr)$_2$]·0.5H_2O_2 and water and N,N-dimethylacetamide adducts. *J. Chem. Res.* **1983**, 302–303.

88. Khodadad, P.; Rodier, N. trans-Diammine-trans-dichloro-trans-dihydroxoplatine(IV) di(peroxyde d`hydrogène). *Acta Crystallogr. Sect. C Cryst. Struct. Commun.* **1987**, *43*, 2219–2220, doi:10.1107/S0108270187088383.

89. Chandrasekaran, A.; Timosheva, N. V.; Day, R.O.; Holmes, R.R. Pseudoheptacoordination and Pseudohexacoordination in Tris(2- N , N -dimethylbenzylamino)phosphane. *Inorg. Chem.* **2002**, *41*, 5235–5240, doi:10.1021/ic020153i.

90. Mak, T.C.W.; Lam, Y.-S. Hexamethylenetetramine oxide–hydrogen peroxide–water (1:1:1). *Acta Crystallogr. Sect. B Struct. Crystallogr. Cryst. Chem.* **1978**, *34*, 1732–1735, doi:10.1107/S0567740878006536.

91. Wang, Y.; Song, S.; Huang, C.; Qi, X.; Wang, K.; Liu, Y.; Zhang, Q. Hunting for advanced high-energy-density materials with well-balanced energy and safety through an energetic host–guest inclusion strategy. *J. Mater. Chem. A* **2019**, *7*, 19248–19257, doi:10.1039/C9TA04677A.

92. Beckmann, J.; Bolsinger, J.; Duthie, A.; Finke, P. New Series of Intramolecularly Coordinated Diaryltellurium Compounds. Rational Synthesis of the Diarylhydroxytelluronium Triflate [(8-Me 2 NC 10 H 6) 2 Te(OH)](O 3 SCF 3). *Organometallics* **2012**, *31*, 238–245, doi:10.1021/om2008259.

93. Andronati, S.A.; Simonov, Y.A.; Chumakov, Y.M.; Gdanets, M.; Bondarev, M.L.; Polishchuk, A.A.; Karaseva, T.L. Synthesis and structure of 1-phenyl-4-(4-phthalimidobutyl)piperazine-1,4-dioxide of dihydroperoxidate. *ZHURNAL Obs. KHIMII* **1996**, *66*, 1736–1740.

94. Shao, M.; Dong, X.U.N.; Tang, Y. Crystal structure investigation of vanadyl complexes of tridentate ligand. (II) - Synthese and crystal structure of 1-(2-pyridylazo)-2-naphtholato-dioxovanadium(V) dimer [$VO_2(C_{15})H_{10}N_3O)]_2(H_2O_2)(CHCl_3)_2$ and pyridine-(1-(2-pyridylazo)-2-naphtolato)peroxo oxovanadium(V) $VO(O_2)(C_{15}H_{10}N_3O)(C_5H_5N)$. *Sci. China Ser. B-Chemistry, Biol. Agric. Med. Earth Sci.* **1988**, *31*, 789–799, doi:https://doi.org/10.1360/yb1988-31-7-789.

95. Farkens, M.; Meyer, T.G.; Neda, I.; Sonnenburg, R.; Müller, C.; Fischer, A.K.; Jones, P.G.; Schmutzler, R. Zur Chemie der l,3,5-Triaza-2-phosphinan-4,6-dione. Teil VI. Darstellung von 1,3,5-Triaza-2 λ3-, 1,3,5-Triaza-2 λ4- und 1,3,5-Triaza-2 λ5-phosphinan-4,6-dionen / Chemistry of the 1,3,5-Triaza-2-phosphinane-4,6-diones. Part VI. Synthesis of 1,3,5-Triaza-2λ. *Zeitschrift für Naturforsch. B* **1994**, *49*, 145–164, doi:10.1515/znb-1994-0201.

96. Olovsson, I.; Templeton, D.H. The Crystal Structure of Hydrogen Peroxide Dihydrate. *Acta Chem. Scand.* **1960**, *14*, 1325–1332.
